# Immunogenomics of Colorectal Cancer Response to Checkpoint Blockade: Analysis of the KEYNOTE 177 Trial and Validation Cohorts

**DOI:** 10.1053/j.gastro.2021.06.064

**Published:** 2021-10

**Authors:** Michele Bortolomeazzi, Mohamed Reda Keddar, Lucia Montorsi, Amelia Acha-Sagredo, Lorena Benedetti, Damjan Temelkovski, Subin Choi, Nedyalko Petrov, Katrina Todd, Patty Wai, Johannes Kohl, Tamara Denner, Emma Nye, Robert Goldstone, Sophia Ward, Gareth A. Wilson, Maise Al Bakir, Charles Swanton, Susan John, James Miles, Banafshe Larijani, Victoria Kunene, Elisa Fontana, Hendrik-Tobias Arkenau, Peter J. Parker, Manuel Rodriguez-Justo, Kai-Keen Shiu, Jo Spencer, Francesca D. Ciccarelli

**Affiliations:** 1 Cancer Systems Biology Laboratory, The Francis Crick Institute, London, United Kingdom; 2 School of Cancer and Pharmaceutical Sciences, King’s College London, London, United Kingdom; 3 Biomedical Research Centre, Guy’s and St. Thomas’ National Health Service Trust, London, United Kingdom; 4 State-Dependent Neural Processing Laboratory, The Francis Crick Institute, London, United Kingdom; 5 Experimental Histopathology, The Francis Crick Institute, London, United Kingdom; 6 Advanced Sequencing Facility, The Francis Crick Institute, London, United Kingdom; 7 Cancer Evolution and Genome Instability Laboratory, The Francis Crick Institute, London, United Kingdom; 8 Cancer Research UK Lung Cancer Centre of Excellence, University College London Cancer Institute, London, United Kingdom; 9 School of Immunology and Microbial Sciences, King’s College London, London, United Kingdom; 10 FASTBASE Solutions S.L, Derio, Spain; 11 Cell Biophysics Laboratory, Ikerbasque, Basque Foundation for Science, Research Centre for Experimental Marine Biology and Biotechnology & Biophysics Institute, University of the Basque Country, Leioa, Bizkaia, Spain; 12 Centre for Therapeutic Innovation, Cell Biophysics Laboratory, Department of Pharmacy and Pharmacology & Department of Physics, University of Bath, Bath, United Kingdom; 13 Medical Oncology, University Hospitals Birmingham National Health Service Foundation Trust, Birmingham, United Kingdom; 14 Drug Development Unit, Sarah Cannon Research Institute UK, London, United Kingdom; 15 Department of Oncology, University College Hospital, London, United Kingdom; 16 Protein Phosphorylation Laboratory, The Francis Crick Institute, London, United Kingdom; 17 Department of Histopathology, University College London Cancer Institute, London, United Kingdom; 18 Department of Gastrointestinal Oncology, University College London Hospital National Health Service Foundation Trust, London, United Kingdom

**Keywords:** Anti-PD1 Immunotherapy, Tumor Mutational Burden, Wnt Signaling, Interferon Gamma, CD8 T cells, A-FRET, amplified Förster resonance energy transfer, B2M, beta-2-microglobulin, CD, cluster of differentiation, CRC, colorectal cancer, DB-CRC, durable benefit colorectal cancer, GzB, granzyme B, IMC, imaging mass cytometry, mIF, multiplexed immunofluorescence, nDB-CRC, no durable benefit colorectal cancer, PD1, programmed cell death 1, PDL1, programmed death-ligand 1, RNA-seq, RNA sequencing, SNV, single-nucleotide variants, T-cell, receptor β-chain sequencing (TCR-seq), TCGA, The Cancer Genome Atlas, TMB, tumor mutational burden, TME, tumor microenvironment, WES, whole-exome sequencing

## Abstract

**Background & Aims:**

Colorectal cancer (CRC) shows variable response to immune checkpoint blockade, which can only partially be explained by high tumor mutational burden (TMB). We conducted an integrated study of the cancer tissue and associated tumor microenvironment (TME) from patients treated with pembrolizumab (KEYNOTE 177 clinical trial) or nivolumab to dissect the cellular and molecular determinants of response to anti- programmed cell death 1 (PD1) immunotherapy.

**Methods:**

We selected multiple regions per tumor showing variable T-cell infiltration for a total of 738 regions from 29 patients, divided into discovery and validation cohorts. We performed multiregional whole-exome and RNA sequencing of the tumor cells and integrated these with T-cell receptor sequencing, high-dimensional imaging mass cytometry, detection of programmed death-ligand 1 (PDL1) interaction in situ, multiplexed immunofluorescence, and computational spatial analysis of the TME.

**Results:**

In hypermutated CRCs, response to anti-PD1 immunotherapy was not associated with TMB but with high clonality of immunogenic mutations, clonally expanded T cells, low activation of Wnt signaling, deregulation of the interferon gamma pathway, and active immune escape mechanisms. Responsive hypermutated CRCs were also rich in cytotoxic and proliferating PD1^+^CD8 T cells interacting with PDL1^+^ antigen-presenting macrophages.

**Conclusions:**

Our study clarified the limits of TMB as a predictor of response of CRC to anti-PD1 immunotherapy. It identified a population of antigen-presenting macrophages interacting with CD8 T cells that consistently segregate with response. We therefore concluded that anti-PD1 agents release the PD1-PDL1 interaction between CD8 T cells and macrophages to promote cytotoxic antitumor activity.

## Graphical abstract

**Figure:**
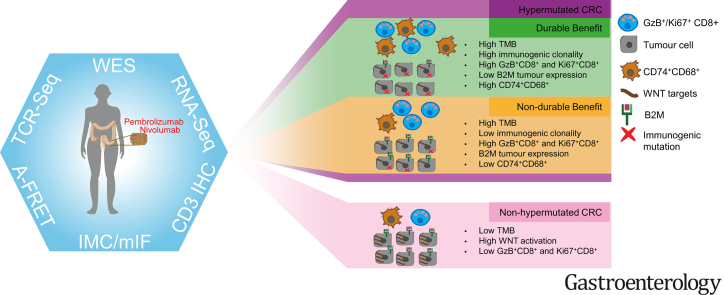

## Teaser

Colorectal cancers responsive to anti-programmed cell death 1 immunotherapy show clonal immunogenic mutations, low Wnt activation, beta-2-microglobulin deregulation, and high infiltration of antigen presenting macrophages interacting with programmed cell death 1-positive cluster of differentiation 8 T cells.

**What You Need to Know**

**Background and Context**

Response of colorectal cancer to immune checkpoint blockade is highly variable, and molecular and cellular determinants of response remain poorly understood.

**New Findings**

Tumor mutational burden is insufficient to predict response in colorectal cancer. Additional predictors are clonal immunogenic mutations, clonally expanded T cells, low Wnt activation, active immune escape, and high CD8 T cells and antigen-presenting macrophage infiltration.

**Limitations**

Due to the restricted use of anti-programmed cell death 1 immunotherapy in hypermutated colorectal cancers, our study has a limited patient cohort size. Additional data from prospective studies are needed.

**Impact**

Colorectal cancer stratification based on tumor mutational burden is limited and may be improved by accounting for other predictors, including the abundance of antigen-presenting macrophages in proximity to CD8 T cells.

Anticancer therapy based on immune checkpoint blockade has driven a paradigm shift in the treatment of several cancer types.^1^ Pembrolizumab and nivolumab, 2 antibodies targeting programmed cell death 1 (PD1) expressed on T cells, have shown efficacy in advanced hypermutated colorectal cancers (CRCs).^2^ Response is thought to depend on rich immune infiltration and high tumor mutation burden (TMB) leading to increased production of peptide neoantigens.^3^ However, despite pervasive tumor immunogenicity, response is highly variable, and approximately half of patients with hypermutated CRCs show no benefit from treatment.^4^

We have dissected the extent to which TMB, cancer dysfunctional genes and pathways, as well as the qualitative and quantitative immune composition of the tumor microenvironment (TME) influence response to immune checkpoint blockade. To reproduce the most common clinical scenario where metastatic biopsies are not routinely taken, we performed a high-dimensional and multiregional profile of primary CRCs or local relapses from 29 patients, divided into a discovery and a validation cohort. The discovery cohort was composed of patients with metastatic disease treated with pembrolizumab as first-line therapy within the KEYNOTE 177 phase III clinical trial^5^ or nivolumab. Most patients did not receive previous treatment, which offered the ideal opportunity to identify critical factors for response to treatment in cancer genetic and transcriptional dysregulation and immune microenvironment composition. We then extended the study to a more heterogenous validation cohort of patients who received anti-PD1 agents alone or in combination and as first-line therapy or in a chemorefractory setting to assess the general validity of our findings.

## Methods

### Patient Populations

Formalin-fixed paraffin-embedded blocks were obtained from surgical resections of the primary tumor or local relapse of 16 patients (UH1–UH16, discovery cohort) and 13 patients (UH17–UH29, validation cohort). UH1 through UH10 were treated with pembrolizumab as part of the KEYNOTE 177 clinical trial (NCT02563002)^5^ and UH11 through UH16 were treated with nivolumab as first-line therapy. UH17 through UH19 were part of the KEYNOTE 177 trial, UH26 received pembrolizumab, UH20 through UH25 and UH29 were treated with nivolumab, UH27 received ipilimumab in combination with nivolumab and then nivolumab alone, and UH28 received nivolumab and then ipilimumab in combination with nivolumab.

Response to therapy was assessed using Response Evaluation Criteria In Solid Tumors 1.1.^6^ Patients were considered to achieve durable benefit if the disease did not progress for at least 12 months after receiving immunotherapy, and no durable benefit if the disease progressed within 12 months. Further details on treatment and other clinical parameters, including tumor staging and prior lines of treatment, are reported as Supplementary Methods and Supplementary Table 1.

### CD3 and H&E Staining

Cluster of differentiation (CD) 3 immunostaining was performed on several slides across the depth of each analyzed tumor block, for a total of 418 regions. Slides were digitally acquired at 20× resolution and loaded into QuPath^7^ to quantify the number of CD3^+^ cells/mm^2^. H&E staining was performed on 13 additional slides of the validation cohort.

### Imaging Mass Cytometry

Imaging mass cytometry (IMC) was performed in 77 regions of the discovery and validation cohorts using 3 panels of 42 antibodies in total. IMC data analysis was done with SIMPLI.^8^ Positive areas for combinations of markers were quantified and normalized over the tissue area or the area of selected immune populations. After segmentation, cell identities were assigned according to the highest overlap with marker-specific masks. Unsupervised cell clustering was performed with Seurat^9^ and used to compare the relative abundances of cell subpopulations between tumor groups. High-density clusters of CD68^+^CD74^+^ cells were identified using DBSCAN.^10^

### Multiplexed Immunofluorescence

Multiplexed Immunofluorescence (mIF) was performed in 24 whole slides of the discovery and validation cohorts using an automated Opal-based mIF staining protocol with 8 antibodies. Fluorescently labeled slides were scanned, and images were loaded into inForm (Akoya Biosciences) for spectral unmixing and autofluorescence isolation.

### Whole-Exome Sequencing

Whole-exome sequencing (WES) was conducted on 32 macrodissected tumor regions and matched normal tissue of the discovery cohort. Sequencing data were aligned using BWA MEM.^11^ Somatic single-nucleotide variants (SNVs) and indels were called using Strelka.^12^ ANNOVAR^13^ was used to annotate exonic or splicing SNVs and indels, and damaging SNVs and indels were identified as previously described.^14^ Copy number analysis was done using ASCAT^15^ and integrated with gene expression data. Amplified genes, deleted genes, heterozygously deleted genes with a damaging mutation in the other allele, and copy number neutral genes with at least 1 damaging mutation were considered as damaged genes. Immunogenic mutations were predicted using Polysolver^16^ and NeoPredPipe,^17^ and their clonality was assessed using PyClone.^18^

### RNA Sequencing

We conducted 3′-RNA sequencing (RNA-seq) on 88 macrodissected regions of the discovery and validation cohorts. Raw reads were processed using the Lexogen QuantSeq 3′ messenger RNA-seq pipeline.^19^ Differential gene expression was assessed using DESeq2.^20^ Pathway enrichment analysis of differential expressed genes was done using MetaCore 20.3 build 70200 (Clarivate Analytics).

### T-Cell Receptor β-Chain Sequencing

T-cell receptor β-chain sequencing (TCR-seq) was performed on 28 macrodissected regions of the discovery cohort. Genomic DNA was submitted to Adaptive Biotechnologies (Seattle, WA) for nonlymphoid tissue (survey level) TCR-seq.^21^ Data were analyzed using the immunoSEQ Analyzer toolset.

### PD1-PDL1 Amplified Förster Resonance Energy Transfer

In situ interaction between PD1 and programmed death-ligand 1 (PDL1) was measured in 58 regions of the discovery cohort via amplified Förster resonance energy transfer (A-FRET)^22^ at FASTBASE Solutions (Derio, Spain). FRET efficiency was calculated from 793 optical fields of view to cover the whole surface of the regions analyzed. The results were expressed as the median fields of view values per region. Detailed protocols and methods are provided in the Supplementary Methods.

## Results

### Response of Hypermutated Colorectal Cancers Is Associated With Clonal Immunogenic Mutations and Clonally Expanded T Cells

**Figure 1:**
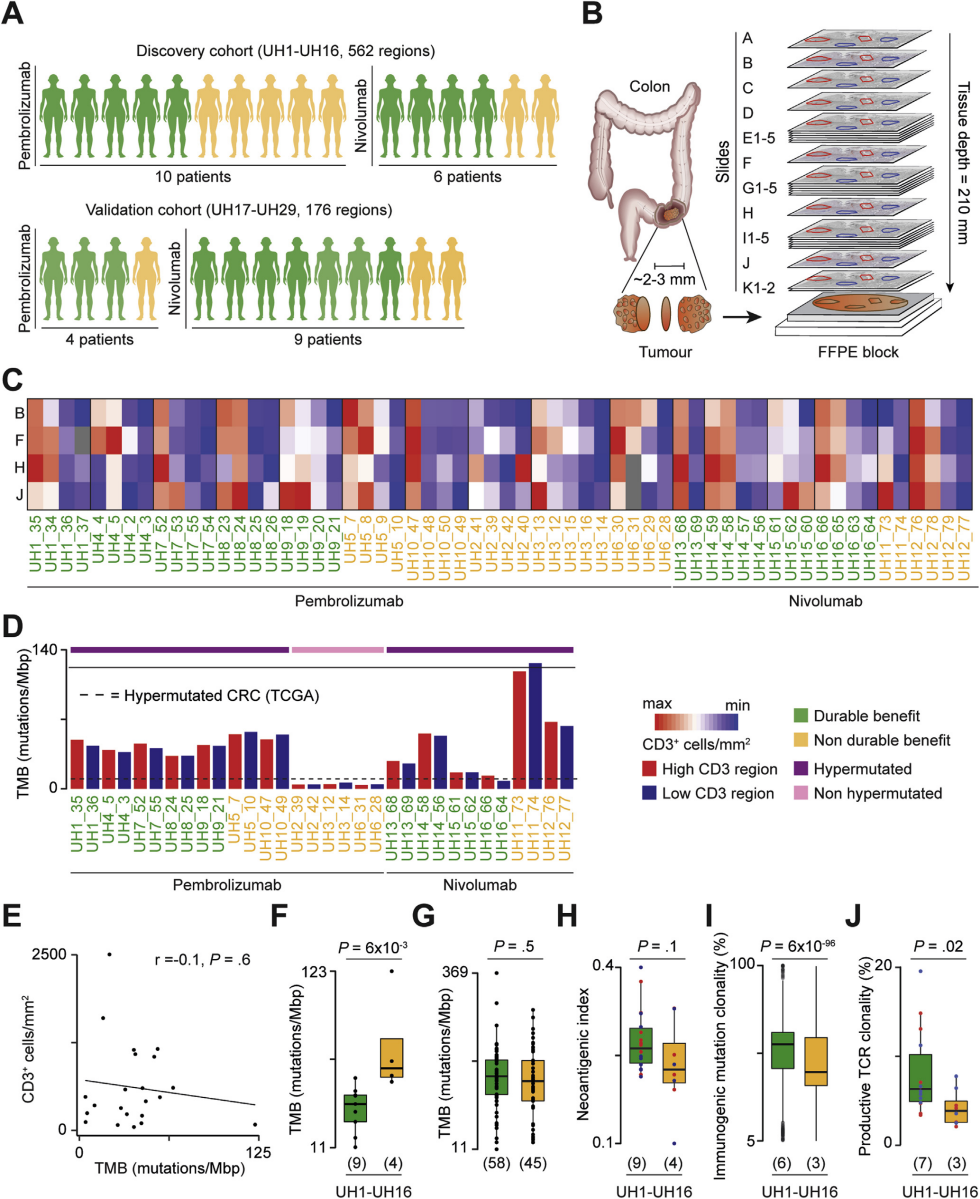
Study design and quantification of tumor heterogeneity. (*A*) Description of the study cohorts. Clinical benefit from the treatment was assessed with Response Evaluation Criteria In Solid Tumors 1.1. (*B*) Experimental design: 24 sequential slides from formalin-fixed paraffin-embedded (FFPE) CRC blocks before treatment were used for multiregional CD3 immunohistochemistry (*slides A, B, F, H*, and *J*), IMC (*slide C*), mIF (*slide D*), WES (*slides E1–E5*), RNA-seq (*slides G1–G5*), TCR-seq (*slides I1–I5*), and A-FRET detection of PD1-PDL1 interaction in situ (*slides K1–K2*). Multiple regions with variable CD3 infiltration were identified in *slide A* and projected to all other slides. (*C*) Quantification of CD3^+^ cells/mm^2^ from immunohistochemistry staining in 60 regions of the discovery cohort using Qupath.^7^ Values were normalized within each patient. The *gray boxes* indicate missing measures. (*D*) TMB of 32 sequenced regions in the discovery cohort. The *dotted line* corresponds to the TMB threshold of hypermutated CRC (12 mutations/megabase pairs).^23^ (*E*) Correlation between CD3^+^ cells/mm^2^ from immunohistochemistry staining of *slide F* (discovery) and *slide E* (validation) and TMB across samples. Average CD3^+^ cell density across multiple regions per slide is reported. For the discovery cohort, TMB was calculated as the average between the 2 sequenced regions. For the validation cohort, TMB was obtained from the FM1 test.^40^ Pearson correlation coefficient *R* and associated *P* value are shown. (*F*) Comparison of TMB between DB- and nDB-CRCs of the discovery cohort and (*G*) in hypermutated CRCs from the validation cohort (Supplementary Table 1) and published studies.^24^, ^25^, ^26^, ^27^, ^28^ For^26^^,^^28^ response was unavailable and the overall survival from the start of immunotherapy was used to define DB (≥12 months) and nDB (<12 months). (*H*) Comparison of neoantigenic index (ratio of predicted immunogenic mutations over all nonsilent mutations) and (*I*) clonality of immunogenic mutations in 17 regions with >30% tumor purity (Supplementary Table 2). Regions with lower purity were excluded because of unreliable mutation clonality assessment.^41^ Results hold true even when using all regions (data not shown). (*J*) Comparison of productive clonality of TCR beta rearrangements between DB- and nDB-CRCs with available data (Supplementary Table 2). The number of patients in each tumor group is reported in *brackets*. Distributions were compared using the 2-sided Wilcoxon’s rank sum test. The *horizontal line* in the middle of each *box* indicates the median; the *top* and *bottom borders* of the box mark the 75th and 25th percentiles, respectively, and the *vertical lines* mark points within 1.5 the inter-quartile range.

To assess how immune infiltration correlates with tumor genetic and transcriptional alterations in CRC, we performed a multiomic and multiregional profile of 24 sequential slides (A–K) from formalin-fixed paraffin-embedded tumor blocks, for a total of 562 regions from 16 patients of the discovery cohort (Figure 1*A*). Ten of these patients received pembrolizumab (UH1–UH10) and 6 nivolumab (UH11–UH16) as a first-line treatment in advanced metastatic setting. According to Response Evaluation Criteria In Solid Tumors 1.1, 9 patients achieved durable benefit, and 7 had no durable benefit from the treatment (Supplementary Table 1). We validated the main findings of the study in 176 additional regions from 13 patients with CRC (UH17–UH29, Supplementary Figure 1*A*) treated with anti-PD1 agents alone or in combinations with other immune checkpoint inhibitors as first-line therapy or in a chemorefractory setting (Supplementary Table 1). Ten of them reached a durable benefit, and 3 had no durable benefit (Figure 1*A*).

Because T cells are the effector cells that mediate the response to anti-PD1 immunotherapy, we selected multiple regions per block with variable T-cell content in proximity to the tumor infiltrating margins (Supplementary Figure 1*B*). These regions were then projected in all sequential slides to perform additional CD3 immunohistochemistry for quantification of T-cell variability in the 3-dimensions of the tumor as well as IMC, mIF, WES, RNA-seq, TCR-seq, and A-FRET detection of the PD1-PDL1 interaction in situ (Figure 1*B*).

As a first analysis, we compared T-cell infiltration between and within tumors (Supplementary Figure 1*C*). In both the discovery (Figure 1*C*) and validation (Supplementary Figure 1*D*) cohorts, we observed widespread intertumor and intratumor heterogeneity of T-cell infiltration, with up to a 38-fold difference in CD3^+^ cell densities between patients and up to a 20-fold difference between regions of the same patient (Supplementary Table 2). To investigate how heterogeneity in T-cell infiltration correlated with TMB, we performed multiregional WES in the discovery cohort by selecting 2 regions per patient, 1 with high and 1 with low T-cell infiltration (Supplementary Table 2). TMB was comparable between regions of the same patient (Figure 1*D*) and did not correlate with T-cell density across samples (Figure 1*E*). Similar lack of correlation was observed in hypermutated CRCs from The Cancer Genome Atlas (TCGA) (Supplementary Figure 1*E*), indicating TMB independence of T-cell heterogeneity.

WES also showed that the TMB in 3 patients (UH2, UH3, and UH6) was lower than 12 mutations/megabase pair (TCGA lower bound of CRC hypermutated phenotype^23^) despite negative MLH1 and PMS2 immunostaining and consistent with resistance to treatment (Supplementary Table 1). All patients of the validation cohort had hypermutated CRCs (Supplementary Table 1).

Given that approximately 50% of patients with hypermutated CRC do not respond to immunotherapy, we compared TMB between hypermutated CRCs with durable benefit (DB-CRCs) and those with no durable benefit (nDB-CRCs) to assess the role of TMB as a marker of response within hypermutated CRC. Surprisingly, in the discovery cohort, DB-CRCs had a significantly lower TMB than nDB-CRCs (Figure 1*F*). When adding hypermutated CRCs from the validation cohort and published studies,^24^, ^25^, ^26^, ^27^, ^28^ we observed no significant difference between DB- and nDB-CRCs (Figure 1*G*). Together with the lack of response in non-hypermutated CRCs (Supplementary Table 1), these results indicate that a TMB below 12 mutations/megabase pair is a predictor of resistance to anti-PD1 immunotherapy in CRC. Above this threshold, TMB is not a predictor of response.

To understand whether the proportion of cancer-associated neoantigens differed between responders and nonresponders, we predicted how many cancer mutations were potentially immunogenic in each patient. In the discovery cohort, the ratio between immunogenic mutations and all mutations (neoantigenic index) was similar between DB- and nDB-CRCs (Figure 1*H*). However, we observed a high number of clonal immunogenic mutations in DB-CRCs (Figure 1*I*), indicating expansion of tumor cells with the same potential immune targets. Similar results were observed using an external data set of hypermutated CRCs treated with immune checkpoint inhibitors^25^ (Supplementary Figure 1*F*). Consistent with dominant antigenic targets, the productive TCR repertoire was also more clonal in DB-CRCs (Figure 1*J*).

Therefore, although hypermutated CRCs responding to anti-PD1 agents do not have more mutations than those failing to respond, they have significantly more clonally expanded immunogenic mutations and T-cell clones.

### Durable-Benefit Colorectal Cancers Show Widespread Immune Dysregulation and Silencing of the Beta-2-Microglobulin Gene

To dissect CRC molecular determinants of response to anti-PD1 agents in CRC, we compared genetic and transcriptional dysregulations between hypermutated and non-hypermutated CRCs as well as between DB- and nDB-CRCs.

**Figure 2:**
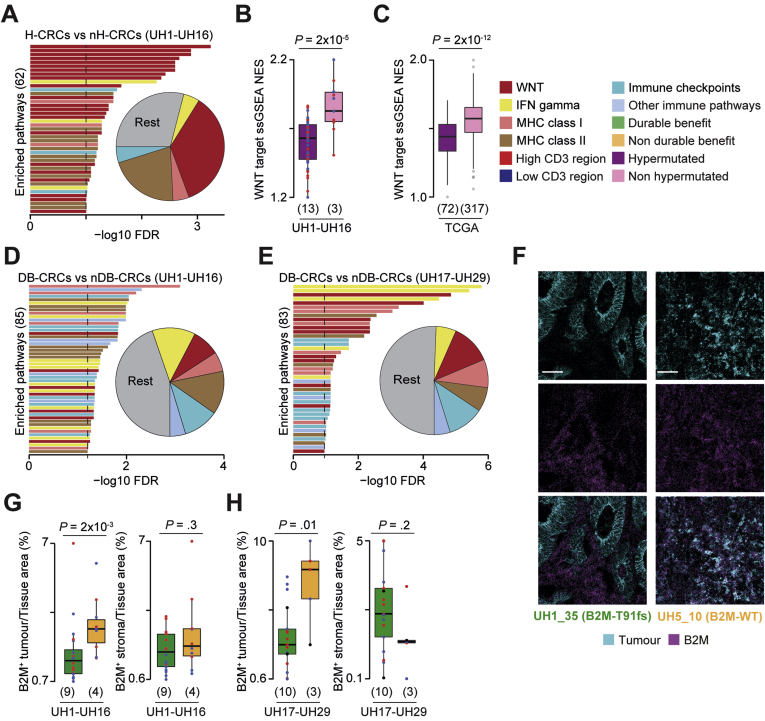
Cancer and immune aberrations across CRC groups. (*A*) Representative enriched pathways in differentially expressed genes between hypermutated and non-hypermutated CRCs of the discovery cohort. The false discovery rate (FDR) was calculated using Benjamini-Hochberg correction. Proportions of immune-related pathways over all enriched pathways are reported as pie chart. Normalized enrichment scores (NES) from single sample Gene Set Enrichment Analysis (ssGSEA)^42^ of 68 transcriptional targets of the Wnt pathway^29^^,^^43^ between hypermutated and non-hypermutated CRCs from (*B*) the discovery cohort and (*C*) TCGA. Representative pathways enriched in differentially expressed genes between DB- and nDB-CRCs from the (*D*) discovery and (*E*) validation cohorts. (*F*) Representative IMC images of CRCs with mutated and wild-type (WT) B2M protein. Scale bar = 50 μm. Comparison of normalized tumor and stroma B2M^+^ areas between DB- and nDB-CRCs of the (G) discovery and (*H*) validation cohorts. Number of patients in each tumor group is reported in brackets. Distributions were compared using the 2-sided Wilcoxon’s rank sum test. IFN, interferon; MHC, major histocompatibility complex. The *horizontal line* in the middle of each *box* indicates the median; the *top* and *bottom borders* of the box mark the 75th and 25th percentiles, respectively, and the *vertical lines* mark minimum and maximum of all the data.

Genes of the Wnt pathway were frequently damaged (Supplementary Figure 2*A*, Supplementary Table 3) and transcriptionally deregulated (Figure 2*A*, Supplementary Table 4) in hypermutated compared with non-hypermutated CRCs. Moreover, Wnt downstream targets were significantly downregulated in hypermutated compared with non-hypermutated CRCs (Figure 2*B*) as confirmed in TCGA (Figure 2*C*). Transcriptional Wnt activation is known to reduce T-cell infiltration,^29^^,^^30^ suggesting a potential impact on the TME composition of these tumors.

Genes encoding members of the interferon gamma pathway, antigen presentation machinery, and other immune-related processes were damaged (Supplementary Figure 2*A*) or transcriptionally dysregulated in hypermutated DB-CRCs compared with nDB-CRCs in the discovery (Figure 2*D*, Supplementary Table 4) and validation (Figure 2*E*) cohorts. Interestingly, 2 DB-CRCs showed a clonal truncating mutation (T91fs) in the beta-2-microglobulin (B2M) gene encoding the invariable subunit of the major histocompatibility complex class I complex (Supplementary Figure 2*B*). Because a B2M antibody was part of the IMC panel (Supplementary Table 5), we could assess that a *B2M* truncating mutation led to no protein expression in the tumor compared with a widespread B2M expression in CRCs with wild-type B2M (Figure 2*F*). In general, B2M protein expression was significantly reduced in the tumor but not in the stroma of DB-CRCs in the discovery (Figure 2*G*) and validation (Figure 2*H*) cohorts as well as in both cohorts combined (Supplementary Figure 2*C*). In melanoma, B2M loss has been associated with resistance to immune checkpoint inhibitors.^31^ Our data indicate an opposite association in CRC, supporting similar recent observations.^32^

### Hypermutated Colorectal Cancers Are Enriched in Cytotoxic and Proliferating CD8 T Cells

**Figure 3:**
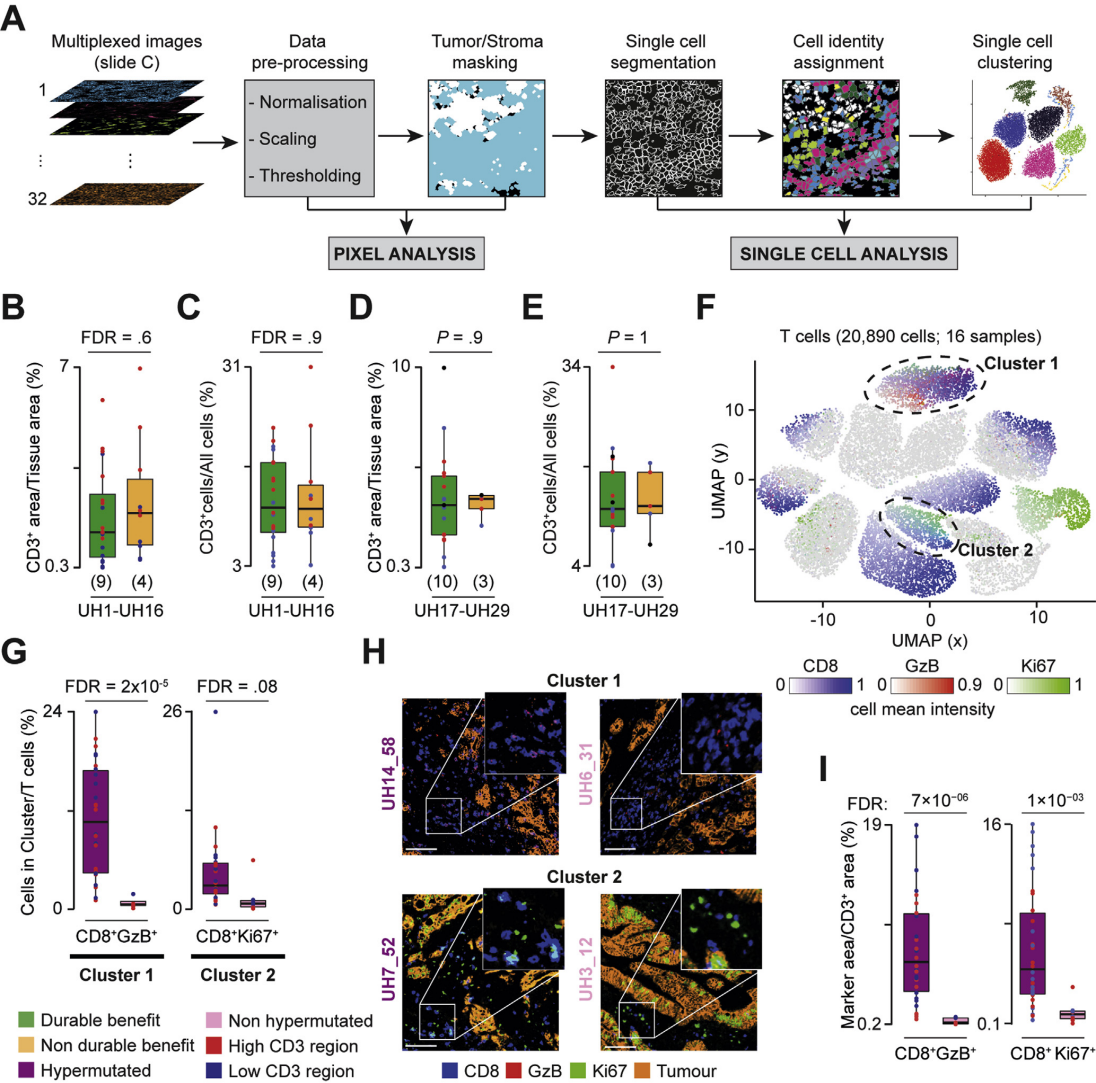
Comparison of T cells infiltrates between CRC groups. (*A*) IMC analysis workflow using SIMPLI.^8^ For each region, images of the markers used (Supplementary Table 5) were preprocessed to extract pixel intensities. Masks for tumor and stroma were derived and used for the pixel analysis. Each region was segmented into single cells that were assigned to tumor or stroma, phenotypically identified through expression of representative markers, and used for single cell clustering. (*B*) Comparison of normalized CD3^+^ areas and (*C*) CD3^+^ cells between DB and nDB-CRCs in the discovery cohort. Benjamini-Hochberg false discovery rate (FDR) correction was applied for testing over 5 immune populations. (*D*) Comparison of normalized CD3^+^ areas and (*E*) CD3^+^ cells between DB- and nDB-CRCs in the validation cohort. (*F*) Uniform Manifold Approximation and Projection (UMAP) map of 20,890 T cells in 38 regions from 16 CRCs of the discovery cohort. Cells were grouped in 13 clusters based on the expression of 12 phenotypic markers using Seurat^9^ (Supplementary Table 8) and colored according to the mean intensities of representative markers. The *circles* indicate the 2 clusters enriched in hypermutated CRCs. (*G*) Proportions of cluster 1 (CD8^+^GzB^+^ cells) and cluster 2 (CD8^+^Ki67^+^ cells) over the total T cells in hypermutated and non-hypermutated CRCs. Distributions were compared using the 2-sided Wilcoxon’s rank sum test. Benjamini-Hochberg FDR correction was applied for testing over 13 clusters. (*H*) IMC-derived images of tumor-associated markers (E-cadherin and pan-keratin) and CD8 and GzB or CD8 and Ki67 in 2 representative samples. *Scale bar* = 100 μm. (*I*) Comparisons of normalized CD8^+^GzB^+^ and CD8^+^Ki67^+^ areas between hypermutated and non-hypermutated CRCs. Distributions were compared using the 2-sided Wilcoxon’s rank sum test. Benjamini-Hochberg FDR correction was applied for testing over 25 combinations of T-cell markers (Supplementary Table 6). The *horizontal line* in the middle of each *box* indicates the median; the *top* and *bottom borders* of the box mark the 75th and 25th percentiles, respectively, and the *vertical lines* mark minimum and maximum of all the data.

To understand the role of TME in the response to anti-PD1 agents, we analyzed multiple tumor regions of the discovery cohort with IMC (Supplementary Figure 3*A*) using markers for T cells, macrophages, neutrophils, dendritic cells, and B cells as well as the tissue structure (Supplementary Table 5). After regional ablation and image processing, we verified that the relative proportion of stroma and tumor cells was similar across samples (Supplementary Figure 3*B*–*F*). We then applied 2 independent and complementary analytical approaches. In one, we compared the normalized pixel area of individual or combined markers (pixel analysis, Figure 3*A*). In the other, we applied single-cell segmentation, assigned cell identities, and compared the relative abundance of immune cell populations identified through unsupervised single-cell clustering (single-cell analysis, Figure 3*A*). Outcomes of all analyses were validated by independent histologic assessment of unprocessed images.

Hypermutated DB- and nDB-CRCs showed no difference in normalized CD3^+^ area (Figure 3*B*, Supplementary Table 6) or proportion of CD3^+^ cells (Figure 3*C*, Supplementary Table 7), confirming that overall T-cell infiltration does not correlate with TMB (Figure 1*E*) or response to therapy. To further investigate whether DB- and nDB-CRCs differed in specific T-cell subpopulations, we performed single-cell clustering using 12 T-cell markers (Supplementary Table 5). We found no qualitative or quantitative differences in T-cell subpopulations between DB- and nDB-CRCs (Supplementary Table 8, Supplementary Figure 4).

Given their relevance to immune checkpoint inhibitors, we further profiled T cells in the validation cohort by adding 5 markers of T-cell function to the 12 used previously (Supplementary Table 5). We confirmed no significant difference in the normalized CD3^+^ area or proportion of CD3^+^ cells between DB- and nDB-CRCs of the validation cohort (Figure 3*D* and *E*). Moreover, single-cell clustering with all 17 phenotypic markers of T cells confirmed no difference in T-cell infiltrates between DB -and nDB-CRCs (Supplementary Table 8).

We repeated the same comparison between hypermutated and non-hypermutated CRCs of the discovery cohort. In this case, we found 2 clusters of CD8 T cells (cluster 1, expressing granzyme B [GzB], and cluster 2, expressing Ki67) significantly higher in hypermutated CRCs (Figure 3*F*–*H*). Pixel analysis confirmed these results (Figure 3*I*, Supplementary Figure 3*G*).

Our analysis identified the cytotoxic and proliferating CD8 T-cell subpopulations that are specifically enriched in hypermutated CRCs, confirming recent reports^33^ and likely due to Wnt low activation observed in these samples (Figure 2*A* and *B*). No qualitative or quantitative differences in any subpopulation of T cells were detected between hypermutated DB- and nDB-CRCs, which were both rich in CD8 T cells.

### Hypermutated Durable Benefit Colorectal Cancers Are Enriched in CD74^+^ Macrophages

To further investigate the association of TME with response, we compared the relative abundance of all other main immune populations between hypermutated and non-hypermutated CRCs or DB- and nDB-CRCs of the discovery cohort.

**Figure 4:**
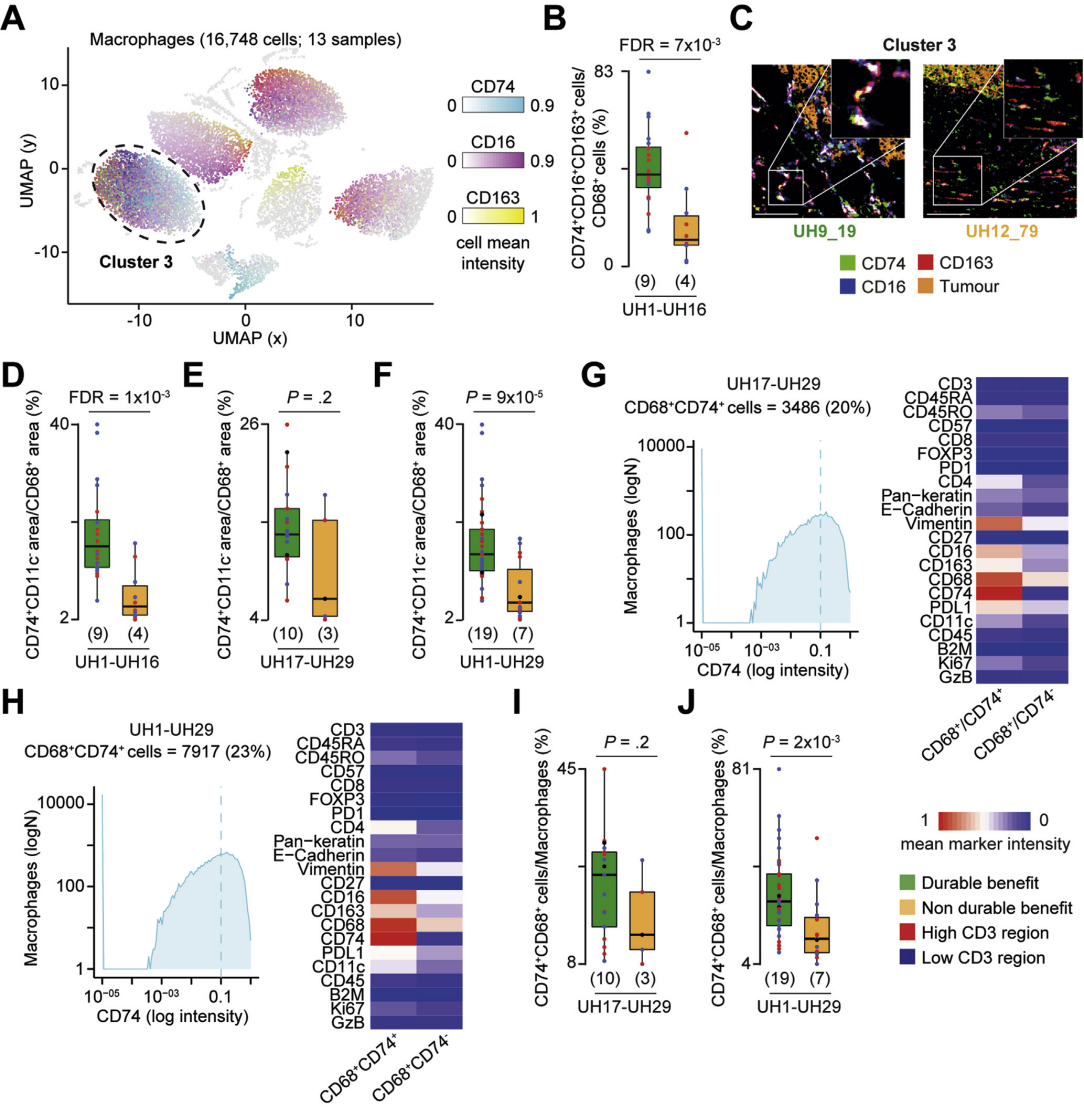
Difference in CD74^+^ macrophages between DB- and nDB-CRCs. (*A*) Uniform Manifold Approximation and Projection (UMAP) map of 16,748 macrophages in 30 regions from 13 hypermutated CRCs in the discovery cohort. Cells were grouped in 9 clusters based on the expression of 11 phenotypic markers using Seurat^9^ (Supplementary Table 8) and colored according to the mean intensities of representative markers. The *circle* indicates the cluster enriched in DB-CRCs. (*B*) Proportions of cluster 3 (CD68^+^CD74^+^ cells) over the total macrophages in DB- and nDB-CRCs. Distributions were compared using the 2-sided Wilcoxon’s rank sum test. Benjamini-Hochberg false discovery rate (FDR) correction was applied for testing over 9 clusters. (*C*) IMC-derived images of CD74, CD16, and CD163 and tumor-associated markers (E-cadherin and pan-keratin) in 2 representative samples. *Scale bar* = 100 μm. (*D*) Comparisons of normalized CD74^+^ area between DB- and nDB-CRCs in the discovery, (*E*) validation, and (*F*) both cohorts using the 2-sided Wilcoxon’s rank sum test. For the discovery cohort, Benjamini-Hochberg FDR correction was applied for testing over 9 combinations of macrophage markers (Supplementary Table 6). (*G*) CD74^+^ macrophages in the validation and (*H*) combined cohorts were identified by applying a threshold of 0.1 CD74 expression to all macrophages after IMC image histologic inspection. Mean marker intensities in CD74^+^ and CD74^−^ macrophages are reported and normalized across all markers and cells. (*I*) Comparison of normalized of CD74^+^ macrophages between DB- and nDB-CRCs in the validation and (*J*) combined cohorts. Distributions were compared using the 2-sided Wilcoxon’s rank sum test. The number of patients in each tumor group is reported in brackets. The *horizontal line* in the middle of each *box* indicates the median; the *top* and *bottom borders* of the box mark the 75th and 25th percentiles, respectively, and the *vertical lines* mark minimum and maximum of all the data.

We found no difference in dendritic cells, neutrophils, and B cells between hypermutated and non-hypermutated CRCs (Supplementary Table 8). However, we observed proportionally higher CD68^+^CD74^+^ cells in DB-CRCs than in nDB-CRCs (cluster 3, Figure 4*A*–*C*), which was confirmed by pixel analysis (Figure 4*D*). To validate these results, we profiled the macrophages also in the validation cohort. Pixel analysis confirmed a higher normalized CD68^+^CD74^+^ area in the validation samples alone (Figure 4*E*) and together with the discovery cohort (Figure 4*F*). To identify CD68^+^CD74^+^ cells, we applied a threshold of 0.1 CD74 expression to all macrophages in the validation cohort (Figure 4*G*) and in all hypermutated CRCs (Figure 4*H*). We verified that CD68^+^CD74^+^ cells identified in this way matched phenotypically to cells in cluster 3 of the discovery cohort (Supplementary Figure 5*A*–*C*). Comparing the proportion of CD68^+^CD74^+^ cells between DB-CRCs and nDB-CRCs we found that it was higher in DB-CRCs of the validation cohort alone (Figure 4*I*) and when all hypermutated CRCs were analyzed together (Figure 4*J*). Therefore, we found that CD68^+^CD74^+^ cells are associated with response to anti-PD1 immunotherapy in CRC.

**Figure 5:**
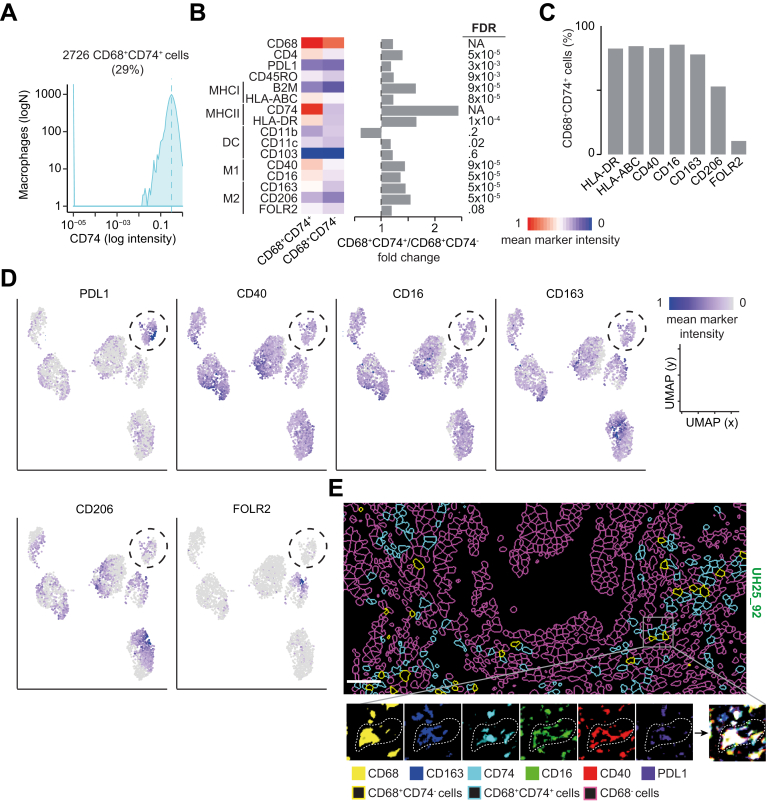
Functional characterization of CD68^+^CD74^+^ cells. (*A*) CD68^+^CD74^+^ cells in 10 DB-CRCs from both cohorts were identified by applying a threshold of 0.35 CD74 expression to all CD68^+^ cells after IMC image histologic inspection. (*B*) Mean marker intensities in CD68^+^CD74^+^ and CD68^+^CD74 cells. Values were normalized across all markers and cells. Marker distributions were compared with the 2-sided Wilcoxon’s rank sum test, and Benjamini-Hochberg false discovery rate (FDR) correction was applied to account for testing over 14 markers. Fold change between the mean expression in CD68^+^CD74^+^ and CD68^+^CD74 cells is reported. DC, dendritic cells. (*C*) Percentage of CD68^+^CD74^+^ cells expressing selected markers associated with antigen presentation and M1 and M2 phenotypes. (*D*) Uniform Manifold Approximation and Projection (UMAP) maps of 2726 CD68^+^CD74^+^ cells in 17 regions from 10 DB-CRCs. Cells were grouped in 6 clusters based on the expression of 16 phenotypic markers using Seurat^9^ and colored according to the mean intensities of representative markers. The *circle* indicates a CPDL1-expressing cluster. (*E*) Single-cell segmentation (*upper panel*) and IMC images (*lower panels*) of selected CD68^+^CD74^+^ cell-associated markers from a representative DB-CRC. The *right bottom panel* reports the combination of all the selected markers. *Scalebar* = 100 μm.

To further characterize these cells, we profiled selected DB-CRCs from both cohorts (Supplementary Table 2) with 16 additional markers (Supplementary Table 5) and identified CD68^+^CD74^+^ cells applying a threshold on CD74 expression (Figure 5*A*). All stained markers, except those associated with dendritic cell functions, were more expressed in CD68^+^CD74^+^ cells than in CD68^+^CD74^−^ cells (Figure 5*B*). HLA-ABC, HLA-DR, CD40, CD16, and CD163 were expressed in >80% of CD68^+^CD74^+^ cells, whereas M2-associated markers, such as CD206 and FOLR2, were specific to smaller subsets (Figure 5*C*). This expression profile suggested that CD68^+^CD74^+^ macrophages may have a T-cell–activating phenotype. Interestingly, approximately 40% of them expressed both M1 and M2 markers, consistent with phenotypic plasticity (Supplementary Figure 5*D*). Single-cell clustering identified 6 distinct groups of CD68^+^CD74^+^ cells, one of which expressed high levels of PDL1, together with CD40, CD16, and CD163, but not CD206 and FOLR2 (Figure 5*D*). Independent histologic inspection confirmed coexpression of these markers in CD68^+^CD74^+^ cells (Figure 5*E*).

Finally, we compared the normalized PD1 or PDL1 protein expression between DB- and nDB-CRCs and found no significant differences in the discovery (Supplementary Figure 6*A*), validation (Supplementary Figure 6*B*), and combined (Supplementary Figure 6*C*) cohorts. This was supported by gene expression analysis (Supplementary Figure 6*D* and *E*) and single-cell clustering, which detected no qualitative or quantitative differences in PD1^+^ or PDL1^+^ cells (Supplementary Table 8). In general, the expression of both PD1 and PDL1 genes was low (Supplementary Figure 6*F*), as confirmed also in TCGA CRCs, where their expression was significantly lower than in melanoma and lung cancer (Supplementary Figure 6*G*). Consistent with their low expression, we could detect PD1-PDL1 protein complex formation in only a minority of regions (Supplementary Table 2), and A-FRET intensity was lower than in melanoma and renal cancer.^22^ The proportion of regions with detectable PD1-PDL1 complex was significantly less in hypermutated than in non-hypermutated CRCs, whereas there was no difference between DB- and nDB-CRCs (Supplementary Figure 6*H*).

Our analyses suggest that a subset of antigen-presenting macrophages with a T-cell– activating phenotype may play a key role in CRC response to anti-PD1 immune therapy. The overall expression of PD1 and PDL1 is low at the gene and protein levels and they show no association with response, indicating that unlike other cancer types,^2^ they are not biomarkers of response in CRC.

### CD74^+^PDL1^+^ Macrophages Interact with PD1^+^ Cytotoxic and Proliferating CD8 T Cells

Our deep investigation of immune infiltrates showed that hypermutated DB-CRCs are immune hot tumors, with high levels of CD74^+^ macrophages compared with nDB-CRCs as well as of cytotoxic and proliferating T cells associated with the hypermutated phenotype. Because CD74^+^ macrophages also expressed PDL1 while the 2 CD8 T-cell populations expressed PD1 (Supplementary Table 8, Supplementary Figure 4), we asked whether these cells were proximal in the TME and interacted through PD1-PDL1 contact.

**Figure 6:**
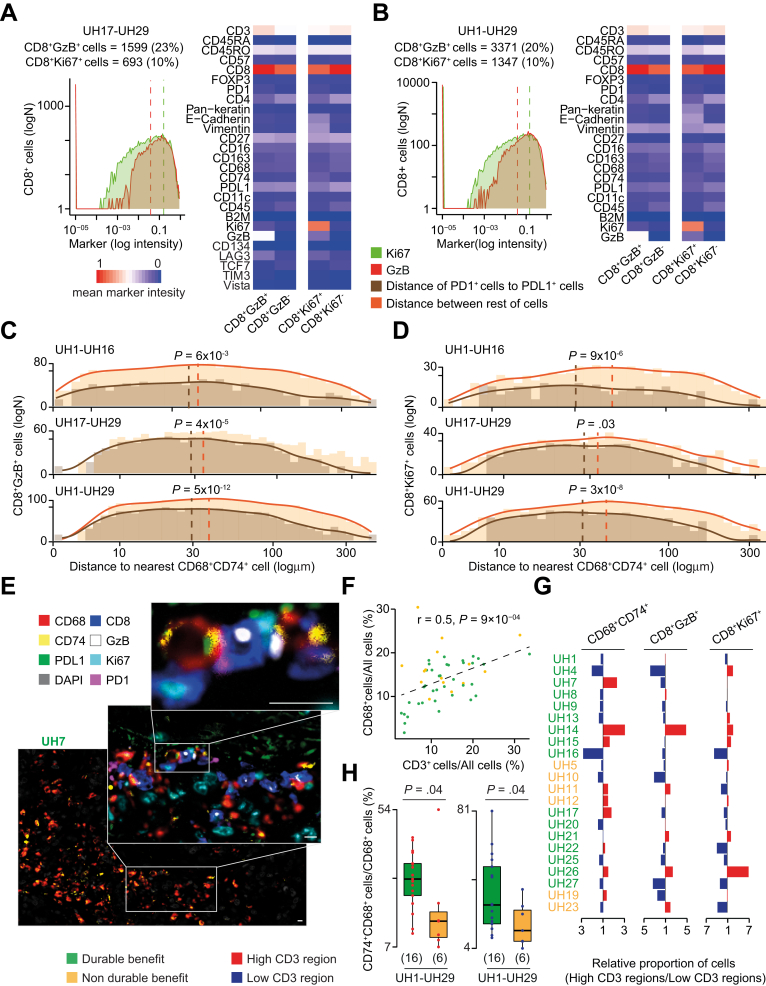
Interaction between CD74^+^ macrophages and GzB^+^Ki67^+^ CD8 T cells. (*A*) CD8^+^GzB^+^ and CD8^+^Ki67^+^ T cells in the validation and (*B*) combined cohorts were identified by applying a threshold of 0.05 GzB and 0.15 Ki67 expression to CD8 T cells, after IMC image histologic inspection. Markers of mean intensities in CD8^+^GzB^+^ or CD8^+^Ki67^+^ and CD8^+^GzB^−^ or CD8^+^Ki67^-^T cells were normalized across all markers and cells. (*C*) Distance distributions of CD8^+^GzB^+^ or (*D*) CD8^+^Ki67^+^ to the nearest CD74^+^ macrophage in the discovery, validation, and combined cohorts. Distances between cells were divided into 1.1-μm bins, and the density curves fitting the histograms were measured. Distributions of PD1^+^ or PDL1^+^ and the rest of the cells were compared using the 2-sided Wilcoxon’s rank sum test. The *dashed lines* represent medians of the distributions. (*E*) High-resolution mIF image of a representative CRC with a highlighted cluster of CD74^+^ macrophages (*main image*) and their interactions with CD8^+^GzB^+^ and CD8^+^Ki67^+^ T cells (*zoom-ins*). The image was scanned at original magnification ×40. *Scale bars* = 10 μm. (*F*) Correlation between normalized T cells and macrophages in 26 DB- and nDB-CRCs of the discovery and validation cohorts. Pearson correlation coefficient *R* and associated *P* value are shown. (*G*) Ratios of normalized CD74^+^ macrophages, CD8^+^GzB^+^, and CD8^+^Ki67^+^ T cells between regions with high and low T-cell infiltration. For samples with more 2 regions, the total cells in the high or low regions were normalized and used to compute the ratio. (*H*) Comparisons of normalized of CD74^+^ macrophages between DB- and nDB-CRCs of the combined cohorts considering only high (*left*) and low (*right*) T-cell infiltration regions. Distributions were compared using the 2-sided Wilcoxon’s rank sum test. The *horizontal line* in the middle of each *box* indicates the median; the *top* and *bottom borders* of the box mark the 75th and 25th percentiles, respectively, and the *vertical lines* mark points within 1.5 the inter-quartile range.

To interrogate this, we identified CD8^+^GzB^+^ and CD8^+^Ki67^+^ cells in the validation cohort (Figure 5*A*) and in all hypermutated CRCs (Figure 6*B*) by applying a threshold of 0.05 (GzB) and 0.15 (Ki67) expression to all CD8 T cells. We verified that these cells were phenotypically similar to clusters 1 (CD8^+^GzB^+^ cells) and 2 (CD8^+^Ki67^+^ cells) of the discovery cohort (Supplementary Figure 7). These 2 populations did not selectively express any additional T-cell markers used in the validation cohort, except the immune checkpoint protein LAG3 (Figure 6*A*). The absence of TCF7 expression in proliferating CD8 T cells suggested that they do not have stem-like characteristics and are not analogous to recently described intratumoral T-cell developmental niches.^34^^,^^35^

After identifying the CD8^+^GzB^+^ and CD8^+^Ki67^+^ T-cell subpopulations, we measured the centroid distance between them and CD68^+^CD74^+^ cells. We then measured the distance between CD8^+^GzB^+^PD1^+^ or CD8^+^Ki67^+^PD1^+^ and CD68^+^CD74^+^PDL1^+^ cells and found that they were closer than to other cells in the discovery, validation, and combined cohorts (Figure 6*C* and *D*). Moreover, a substantial fraction of CD74^+^ macrophages (52% in DB-CRCs and 32% in nDB-CRCs) aggregated in high-density clusters composed of ≥5 cells/10,000 μm^2^^34^. These computationally identified clusters of CD74^+^ macrophages also contained CD8^+^GzB^+^ and CD8^+^Ki67^+^ cells (Supplementary Figure 8). The existence of these clusters was confirmed through independent histologic inspection (Supplementary Figure 9), which also detected direct interactions between CD8^+^GzB^+^PD1^+^ or CD8^+^Ki67^+^PD1^+^ and CD68^+^CD74^+^PDL1^+^ cells. To confirm these interactions at higher resolution, we performed mIF with 8 key markers defining CD74^+^CD68^+^, GzB^+^CD8^+^, and Ki67^+^CD8^+^ cells (Supplementary Table 5). We confirmed the presence of clusters of CD74^+^ macrophages in close proximity to CD8^+^GzB^+^ and CD8^+^Ki67^+^ T cells and detected their interaction via PD1-PDL1 contact (Figure 6*E*, Supplementary Figure 10).

Taking advantage of the multiregional profiles, we asked how the observed intratumor T-cell heterogeneity (Figure 1*C*, Supplementary Figure 1*D*) affected the distinctive infiltration pattern of DB-CRCs. First, we observed that tumor regions rich in T cells were also rich in macrophages (Figure 6*F*), indicating that intratumor heterogeneity involves a more general pattern of coinfiltration. Next, we investigated how the 3 key populations of DB-CRCs (CD8^+^GzB^+^, CD8^+^Ki67^+^, and CD68^+^CD74^+^ cells) were distributed across regions of the same tumor. We observed that their relative proportions were highly variable between high and low infiltrate regions and that no clear pattern could be seen discriminating DB- and nDB-CRCs (Figure 6*G*). Despite such a heterogeneous composition of the immune infiltrates, we observed consistently higher proportion of CD74^+^ macrophages in DB-CRCs than in nDB-CRCs independently of T-cell infiltration levels (Figure 6*H*).

Our data consistently indicate that CD74^+^ macrophages differ between DB- and nDB-CRCs across cohorts and regions. We therefore propose that their interaction with CD8^+^GzB^+^PD1^+^ and CD8^+^Ki67^+^PD1^+^ cells through PDL1 is key to confer durable benefit from treatment.

## Discussion

In this study, we integrated multiregional genomic, transcriptomic, histopathologic, and immune-phenotypic data to characterize the tumor-immune interactions determining response of CRC to immune checkpoint blockade.

After extensive unsupervised investigation of variability in leukocyte subpopulations between hypermutated DB- and nDB-CRCs using multiple approaches, we found CD74^+^ macrophages were the only immune cell population that consistently segregated with response in DB-CRCs. This is remarkable, given the observed genetic and immune inter- and intratumor heterogeneity and the diversified treatment history and suggests that CD74^+^ macrophages could be further developed as a robust predictor of response in a broad range of patients. These macrophages express PDL1 and are in close proximity to PD1^+^ CD8 T cells, indicating that the PD1/PDL1 interaction between these cells may restrain CD8 T-cell function and may be the one that anti-PD1 antibodies break to release cytotoxic antitumor activity.

The high cytotoxic CD8 infiltration in hypermutated CRCs is likely enabled by the low activation of the Wnt pathway, resulting in an immune hot environment. To evade immune elimination, hypermutated DB-CRCs develop immune escape mechanisms via genetic inactivation or transcriptional repression of antigen-presenting genes. Interestingly, unresponsive hypermutated CRCs do not show such a pervasive disruption of the antigen presentation machinery, despite comparably high levels of CD8 infiltration. The molecular mechanisms by which these tumors survive the attack of cytotoxic CD8 T cells need further investigation, although a possible explanation could reside in their significantly reduced proportion of CD74^+^ macrophages.

Similarly, further investigations are required to explain how tumors lacking B2M can respond to immunotherapy. In B2M-null CRC mice, response to anti-PD1 agents relies on CD4 T cells rather than CD8 T cells.^36^ Although we did not observe any difference in CD4 T cells between DB- and nDB-CRCs, this suggests that anti-PD1 agents may act through several mechanisms, including antigen-independent T-cell activation or reinduction of B2M expression.

Our study also highlights cancer-specific traits of response to anti-PD1 immunotherapy. We show that in CRC, high TMB is necessary but not sufficient to achieve durable benefit and that above the critical threshold of the hypermutated phenotype, even CRCs with very high TMB may not respond to treatment. This is different from lung cancer and melanoma, where response always positively correlates with TMB.^37^^,^^38^ In CRC, a low TMB is a marker of resistance, not because of a low neoantigenic load but because it is associated with a higher activation of the Wnt pathway leading to immune cold tumors.

Moreover, while the impairment of antigen presentation in immune hot tumors is shared across cancer types,^39^ the association of B2M loss with response and the overall low PD1 and PDL1 expression are specific traits of CRC. This suggests that universal predictors of response to immunotherapy may not exist and that the specific genetics of the tumor as well as the features of the TME should be considered. In the case of CRC, these may include clonal immunogenic mutations and expanded T cells, low activation of the Wnt pathway, and high infiltration of CD8 T cells coupled with CD74 macrophages.
