# Supplementary material for: Immunogenomics of Colorectal Cancer Response to Checkpoint Blockade: Analysis of the KEYNOTE 177 Trial and Validation Cohorts
Source: Gastroenterology. 2021 Oct;161(4):1179–93. doi: 10.1053/j.gastro.2021.06.064 (PMC8527923; doi:10.1053/j.gastro.2021.06.064)
Supplement: Supplementary Material [file mmc1.pdf]

Supplementary Methods

Patient cohorts, treatment and experimental design

FFPE blocks were obtained from the resection of the primary tumour or local relapse of 16 patients (discovery cohort) and 13 patients (validation cohort) treated with immune checkpoint inhibitors in the setting of metastatic CRC until disease progression, unacceptable toxicity or completion of treatment (Supplementary Table 1). In the discovery cohort, patients UH1-UH10 were treated with Pembrolizumab (200 mg every three weeks) as part of the KEYNOTE 177 clinical trial (ClinicalTrials.gov, NCT02563002)[1], while patients UH11-UH16 were treated with Nivolumab (240mg every two weeks). In the validation cohort, patients UH17-UH19 were part of the KEYNOTE 177 trial, UH26 received Pembrolizumab (2mg/kg every three weeks) and patients UH20-UH25 and UH29 were treated with Nivolumab (240 mg every two weeks). Patient UH27 received Ipilimumab (1mg/kg) in combination with Nivolumab (3mg/kg) every three weeks for four cycles followed by Nivolumab alone (240 mg every two weeks). Patient UH28 received Nivolumab (3mg/kg) for two cycles, then Ipilimumab (1mg/kg) in combination with Nivolumab every three weeks for three cycles. Patients treated with Nivolumab were enrolled in the UK wide Bristol Myers Squibb Individual Patient Supply Request Programme as per Article 5/1 of Article Directive 2001/83/EC. All patients were consented at the UCL Cancer Institute Pathology Biobank - REC reference 15/YH/0311.

Response to therapy was assessed using the formal Response Evaluation Criteria in Solid Tumours (RECIST)[2] version 1.1. Patients were considered to achieve durable benefit (DB) if the disease did not progress for at least 12 months after commencing immunotherapy; no durable benefit (nDB) if the disease progressed within 12 months. Twelve-month cut-off was considered clinically better than the progression-free survival from chemotherapy as first line treatment of metastatic stable (8.3 months[3]) or hypermutated (8.2 months[1]) CRC.

Twenty-four sequential sections were cut from each FFPE tumour block of samples UH1-UH16 using a microtome. Sections were then used for CD3 staining (slides A, B, F, H and J); Imaging Mass Cytometry (IMC, slide C); multiplexed Immunofluorescence (mIF, slide D); Whole Exome Sequencing (WES, slides E1-5); RNA sequencing (RNA-seq, slides G1-5); T-Cell Receptor Beta sequencing (TCR-seq, Slides I1-5) and to detect PD1-PDL1 interaction *in situ* (slides K1-2). For samples

UH17-UH23 and UH25-UH27, 11 sequential sections were used for CD3 staining (slides A, E and G); Haematoxylin and Eosin (HE) staining (slide B); IMC (slide C); mIF (slide D) and RNAseq (slides F1-5). Since UH24, UH28 and UH29 were biopsies, RNAseq could not be performed and only four sequential sections were used for CD3 and HE staining (slides A and B); IMC (slide C) and mIF (Slide D). Sections used for CD3 and HE staining, IMC, mIF and A-FRET were 4μm thick, while those used for DNA and RNA extraction were 10μm thick. Tumour content was assessed by a board-certified surgical pathologist (M.R.J.).

CD3 staining and quantification

CD3 staining was performed upon slide dewaxing and heat-induced epitope retrieval (HIER) using Antigen Retrieval Reagent-Basic (R&D Systems). Tissues were blocked and incubated first with anti CD3 antibody (Dako, Supplementary Table 5) and subsequently with horseradish peroxidase conjugated anti rabbit antibody (Dako). They were then stained with 3,3' diaminobenzidine (DAB) substrate (Abcam) and haematoxylin. Slide A was reviewed by a certified pathologist (M.R-J) to identify two to four regions per slide with variable $CD3^+$ infiltration (for a total of 90 regions, Supplementary Table 2) in proximity to the invasive margins of the tumour (Supplementary Figure 1B).

Digital acquisition of CD3 stained slides was performed using Hamamatsu Nanozoomer (Hamamatsu Photonics) or Axioscan Z1 (Zeiss) at 20x resolution. The whole slide images were then loaded into QuPath[4] v0.2.0-m4 to quantify $CD3^+$ infiltration within each region. The “Estimate Stain Vector” function was run as pre-processing step to increase the contrast between DAB and haematoxylin. The outlines of the regions delimited by the pathologist in slide A and projected in all other slides (Supplementary Figure 1C). The regions were then divided into 0.09 mm^2 large tiles and $CD3^+$ cells were quantified within each tile using the “positive cell detection” function. The median value of $CD3^+$ cells per mm^2 across all tiles was considered as representative of $CD3^+$ infiltration for that region. For slides B and I, $CD3^+$ cells were also quantified for the whole tumour region.

Imaging mass Cytometry (IMC)

Three panels of 42 antibodies in total were assembled to represent the main immune and stromal populations of the gut TME (IMC panels I, II and III,

Supplementary Table 5). Twenty-five of these antibodies were already metal-tagged (Fluidigm), while 17 were purchased in a carrier-free form, tested via immunohistochemistry and tagged using the Maxpar X8 metal conjugation kit (Fluidigm). To identify the optimal dilution for each antibody, concentrations ranging from 1/50 to 1/5,000 were tested in FFPE appendix sections. After staining and ablation, images were visualised using MCD Viewer (Fluidigm) and the concentration giving the best signal to background ratio was selected (Supplementary Table 5).

IMC was performed in 38 regions of the discovery cohort using IMC panel I, in 22 regions of the validation cohort using IMC panel II, and in additional 17 regions of selected samples from both cohorts with IMC panel III (Supplementary Table 2). In the discovery cohort, the two regions (one with low and one with high CD3 infiltration) with the highest tumour content were selected except for UH4 UH6, UH9 and UH12. For UH6, UH9 and UH12, all four regions were analysed, for UH4, the two high and two low CD3 regions were analysed together, to be consistent with WES and RNA-seq analyses (see below). In the validation cohort, the two regions with the highest difference in CD3 infiltration were selected except for the three biopsies (UH24, UH28 and UH29) and UH18, where only one region was analysed. Slides were incubated for one hour at 60°C, dewaxed, rehydrated and subjected to HIER using a pressure cooker and Antigen Retrieval Reagent-Basic (R&D Systems). Tissues were blocked in a solution containing 10% BSA (Sigma), 0,1% Tween (Sigma), 1:50 Kiovig (Shire Pharmaceuticals) Superblock Blocking Buffer (Thermo Fisher) for two hours at room temperature. The primary antibody mix was prepared in blocking solution at the selected concentration for each antibody and incubated overnight at 4°C. Slides were then washed twice in PBS and PBS-0.1% Tween and incubated with 2 isotopes (^{191}Ir and ^{193}Ir) of DNA intercalator Cell-ID™ Intercalator-Ir (Fluidigm) 1.25mM diluted in PBS for 30 minutes at room temperature. Slides were then washed once in PBS and once in MilliQ water and air-dried. Stained slides were loaded in the Hyperion Imaging System (Fluidigm) imaging module to obtain light-contrast high resolution images of approximately 4 mm^2. For each region, a 1 mm^2 area with high tumour content and representative of the median CD3$^+$ content of the region was selected for laser ablation (Supplementary Figure 3A) at 1 μm/pixel resolution and 200Hz frequency.

IMC pixel analysis

IMC data analysis was performed with SIMPLI[5] as summarised in Figure 3A. For each of the 77 ablated regions, TIFF images from each antibody and two DNA intercalators were obtained from the raw IMC .mcd and .txt files using imctools[6]. Pixel intensities for each channel were normalised to the 99th percentile of the intensity distribution and the obtained values scaled between 0 and 1. Background pixels were removed using global thresholding with CellProfiler[7] 3.1.8. After visual inspection, channels for PD1, PDL1, GzB, CD45RA, TIM3, Vista, TCF7, CD134, CD206 and FOLR2 were further filtered using probability masks produced with Ilastick[8] 1.3.0. For this purpose, random forest classifiers were trained using closely related markers (CD3 for PD1; Vimentin for PDL1; CD8 and CD15 for GZB; CD45 and CD45RO for CD45RA; CD68 for FOLR2 and CD206). The resulting background probability masks were converted into binary images with CellProfiler[7] 3.1.8 and applied to the original normalised images to remove the background. Custom R scripts were used to count the positive pixels in all processed images for each channel. The sum of all positive pixels for a channel constituted the positive area for that channel. Given B2M low expression, an ad-hoc threshold was applied on the normalized intensity and all pixels higher than 0.5 were considered as positive. For regions UH19_87, UH27_96, and UH27_97, the CD3 masking threshold was adjusted after manual inspection to 0.175, 0.15 and 0.15 respectively.

Tumour masks were generated with CellProfiler[7] 3.1.8 summing up the Pan-keratin and E-cadherin channels for all regions except UH18_103 and UH22_112, where only E-cadherin was used. The resulting images were smoothened with a Gaussian filter and filling up all <30 pixel negative areas. The stroma masks were obtained using the Vimentin, SMA and DNA channels in the discovery cohort and Vimentin, CD68, CD11c, CD3, CD27 and CD45 channels in the validation cohort. All <20 pixel negative areas were filled up. The tissue mask for each region corresponded to the sum of tumour and stroma masks. Pixel analysis was performed by normalising the positive areas for each marker or combination of markers over the total tissue area or the area of the five main immune populations (T cells, B cells, macrophages, dendritic cells and neutrophils) for the discovery cohort and for T cell and macrophages only for the validation cohort (Supplementary Table 6).

IMC single cell analysis

Single cell analysis was based on cell segmentation, assignment of cell identity and phenotype clustering (Figure 3A).

Cell segmentation was performed with CellProfiler[7] 3.1.8 identifying nucleus and membrane of each cell in each region. First, the two DNA channels were multiplied and used for nucleus segmentation using local Otsu thresholding. Second, all channels for the membrane markers (CD3, CD20, CD27, CD16, CD11c, CD15, SMA, CD34, Vimentin and Pan-keratin for UH1-UH16 and CD45, Pan-keratin and E-cadherin for UH17-UH29) were used to obtain membrane images. Cell masks were then generated by radially expanding each nucleus up to 10 pixels on the membrane mask and only cells overlapping with the tissue mask were retained. Finally, the mean intensity of all markers was measured in each cell in each region.

Cell identities were assigned according to the maximum overlap of the cell area with marker-specific thresholds identified by the histologist (J.S.) after image manual inspection. These thresholds were: ≥25% of CD3$^+$ mask for T cells; ≥10% of CD11c$^+$ CD68- mask for dendritic cells; >10% of the sum of CD68$^+$ CD11c$^+$ and CD68$^+$ CD11c- masks for macrophages; ≥5% of IgA$^+$, IgM$^+$, CD20$^+$, and CD27$^+$ mask for B cells; and ≥25% of CD15$^+$ mask for neutrophils. Cells that did not overlap with any of these markers were defined as tumour cells if they overlapped ≥80% with the tumour mask or were left unassigned otherwise. Within CD3$^+$ cells, PD1$^+$ cells were identified as those showing ≥1% overlap with the PD1 mask. PDL1$^+$ cells were identified as those overlapping ≥10% of the PDL1 mask.

For the discovery cohort, single cell phenotype clustering was performed for T cells, B cells, macrophages, dendritic cells, neutrophils, PD1$^+$ and PDL1$^+$ cells separately using Seurat[9] 2.4 with custom R scripts for IMC data analysis. Independent clustering was used to compare the relative abundance of cell subpopulations between hypermutated and non-hypermutated CRCs or DB- and nDB-CRCs using Pembrolizumab and Nivolumab samples alone or combined. The total number of cells used in each clustering is shown in Supplementary Table 7. For each main population, the clustering was based on the mean expression of a set of markers typical of that population (Supplementary Table 5). The mean marker intensities across all cells were integrated using multiple canonical correlation analysis (CCA) and aligning the CCA subspaces to reduce the inter-sample variability. The resulting CCA vectors were then used as input for unsupervised clustering with ten values of resolution, ranging from 0.1 to 1.0. The ten resulting sets of clusters were manually inspected and the one with

the highest number of biologically meaningful clusters was chosen. For the validation cohort, T cells, macrophages, $PD1^+$ and $PDL1^+$ cells were identified as described above. $CD74^+$ macrophages and $CD8^+GzB^+$ $CD8^+Ki67^+$ T cells were identified using specific expression thresholds on the mean cell intensity (0.1 for CD74; 0.1 for CD8; 0.05 for GzB; 0.15 for Ki67). $CD8^+$ T cells positive for both the Ki67 and the GzB threshold were identified as $CD8^+GzB^+$ or $CD8^+Ki67^+$ T cells according the marker with the highest intensity. T cells in the validation cohort underwent single cell clustering, using all 17 T cell markers (Supplementary Table 5). The distribution of cells within each cluster over the total cells was compared between DB- and nDB-CRCs or hypermutated and non-hypermutated CRCs using two-sided Wilcoxon rank sum test, correcting for FDR. All comparisons are shown in Supplementary Table 8.

For the 17 regions stained with IMC panel III, T cells, macrophages, dendritic cells and tumour cells were identified as described above (Supplementary Table 7). $CD74^+$ macrophages were identified using a threshold of 0.35 on the mean cell intensity. Single-cell clustering of the identified $CD74^+$ macrophages was performed using using Seurat[9] 2.4 with 16 macrophage markers (Supplementary Table 5).

IMC neighbour and cluster density analysis

The pixel coordinates of the centroid of each cell were extracted from the cell masks with CellProfiler[7] 3.1.8 and used to measure the Euclidean distances between each pair of cells in each region. High-density clusters of $CD68^+CD74^+$ within each region were identified using DBSCAN (Density-Based Spatial Clustering of Applications with Noise[10]) as implemented in the fpc R package version 2.2.5. Starting form cell pixel coordinates, highly dense clusters were defined as portions of the ablated regions with ≥5 $CD68^+CD74^+$per 10,000μm^2, corresponding to a minimum number of five points (MinPts) within a radius (eps) of 56.42µm.

Multiplexed Immunofluorescence (mIF)

mIF was performed on slide D of 24 DB- and nDB samples (Supplementary Table 2). An automated Opal-based mIF staining protocol was developed using a Ventana Discovery Ultra automated staining platform (Roche) with eight markers specific for $CD8^+GzB^+PD1^+$, $CD8^+Ki67^+PD1^+$ and $CD68^+CD74^+PDL1^+$ cells, DAPI and Opal fluorophores (Supplementary Table 5). Antibody dilution, incubation time and effect of denaturation steps as well as Opal dilution were assessed for each marker

following manufacturer's instructions. The optimal antibody-Opal pairing was achieved considering the expected expression and cellular localisation of each marker and the fluorophore brightness to minimize fluorescence spillage. The final staining order was CD74, TCF7, PDL1, Ki67, PD1, GzB, CD68 and CD8.

Slides were baked for 1 hr at 60°C, loaded onto the autostainer and subjected to a fully automated staining protocol involving deparaffinisation (EZ-Prep solution, Roche), HIER (DISC. CC1 solution, Roche) and seven sequential rounds of 1 hr incubation with the primary antibody, 12 minutes incubation with the HRP-conjugated secondary antibody (DISC. Omnimap anti-Ms HRP RUO or DISC. Omnimap anti-Rb HRP RUO, Roche) and 16 minute incubation with the Opal reactive fluorophore (Akoya Biosciences). For the last round of staining, tissues were incubated with Opal TSA-DIG reagent (Akoya Biosciences) for 12 minutes and with Opal 780 reactive fluorophore for 1 hour (Akoya Biosciences). Before each round of staining, a denaturation step (100°C for 8 minutes) was introduced to remove the primary and secondary antibodies from the previous cycle without disrupting the fluorescent signal. Once the staining was completed, the slides were counterstained with 4',6-diamidino-2-phenylindole (DAPI, Akoya Biosciences) and coverslipped using ProLong Gold antifade mounting media (Thermo Fisher Scientific). Fluorescently labelled slides were scanned using a Vectra Polaris automated quantitative pathology imaging system (Akoya Biosciences). Spectral libraries were constructed with the inForm 2.4 image analysis software (Akoya Biosciences) following the manufacturer's instructions. Whole-slide scans were obtained at 20x and 40x magnification using appropriate exposure times, and several fields of views were selected per slide and loaded into inForm (Akoya Biosciences) for spectral unmixing and autofluorescence isolation using the spectral libraries.

DNA sequencing

All regions were macro-dissected with a needle under a stereo microscope using slide A as a guide (Supplementary Figure 1G). Genomic DNA was extracted from 32 tumour regions and 16 matched normal tissue of slides E1-5 of samples UH1-UH16 (Supplementary Table 2) and the two regions corresponding to those used for IMC were selected. For UH4, the two high and low $CD3^+$ regions were merged to obtain enough DNA for library preparation. DNA was extracted using GeneRead DNA FFPE kit (Qiagen) and DNA libraries were prepared using 50-200ng of genomic DNA with

the KAPA HyperPrep kit (Roche). Protein-coding genes were captured using SureSelectXT Human All Exon V5 probes (Agilent) and sequenced on Illumina HiSeq 4000 using 100bp paired end read protocol, according to manufacturer's instructions. Approximately 100 million reads were generated per sample.

Raw reads were aligned to GRCh38 reference human genome using BWA MEM[11] v0.7.15 after pre-alignment quality control. Regions harbouring small insertions and deletions (indels) were re-aligned locally using GATK[12] v3.6. The resulting BAM files were sorted, merged, marked for duplicates and subjected to post-alignment quality control using Picard v2.10.1. The final mean depth of coverage was >70x for tumour and >30x for normal samples, considering only targeted exons as defined in the SureSelectXT BED file (50.5Mbp in total).

Somatic SNVs and indels were called using Strelka[13] v2.9.0 on the targeted exome extended 100bp in both directions. Mutations were retained if they had an Empirical Variant Scoring (EVS) >7 for SNVs and >6 for indels in at least one region of the same patient. Mean sensitivity in variant calling was >91% in all patients expect UH16 (33%) as assessed using 241 somatic mutations from FM1[14] or the patient pathological reports for comparison. Nineteen mutations in FM1 or pathological reports but missed by Strelka were added to the pool of somatic alterations after manual check.

For samples UH1-UH3, UH7-UH10 and UH12, copy number analysis was done using ASCAT[15] v2.5.2. To process WES data, AlleleCount[16] v4.0.0 was run on germline SNPs from 1000 Genomes Phase 3[17] after correction for GC bias. For each SNP, a custom script was used to calculate the LogR and B-Allele Frequency (BAF). SNPs with <6 reads were filtered out in all samples except UH1 and UH10 where 5 or 7 reads were used. Because of degraded starting DNA of samples UH4-UH6, UH11 and UH13-UH16, the DepthOfCoverage option of GATK[12] v3.6 was used to calculate SNP LogR and copy numbers for genomic segments were obtained using Copynumber R package. The gene copy number was derived from that of the genomic segment covering at least 25% of the gene length.

Prediction of damaged genes and immunogenic mutations

ANNOVAR[18] (release 16/04/2018) was used to annotate exonic or splicing SNVs and indels. All truncating mutations (stop-gains, stop-losses and frameshift indels) were considered as damaging. Non-truncating mutations (non-frameshift indels

and missense SNVs) were considered damaging if predicted by at least five function-based methods or two conservation-based methods[19]. Mutations within two bps of a splicing junction were considered as damaging if predicted by at least one ensemble algorithm in dbNSFP. Gain of function mutations were predicted using OncodriveClust[20] for the discovery patients together, with default parameters and with false discovery rate (FDR) <10%.

A gene was considered amplified if its copy number was >1.4 times the sample ploidy or >2 if the ploidy was not available in both regions of the same patient, or if its CPM was >1.5 than in the other region of the same patient. A gene was considered as deleted if it had copy number = 0, CPM = 0 and had no mutations. A gene was considered deleted in heterozygosity if it had copy number = 1.

Amplified genes, deleted genes, heterozygously deleted genes with a damaging mutation in the other allele and copy number neutral genes with at least one damaging mutation were considered as damaged genes.

To predict putative immunogenic mutations from all somatic SNVs and indels, HLA typing of each patient was predicted from the normal BAM files using Polysolver[21] v4 (Supplementary Table 9). NeoPredPipe[22] was then used to predict neoantigens in expressed genes (CPM >0), with a strong HLA binding (rank <0.5%) and cross-referenced with known epitopes (UniProt reference proteome). SNVs or indels generating at least one neoantigen were considered as potentially immunogenic. The neoantigenic index was calculated for each region as:

$$Neoantigenic\ index = \frac{number\ of\ immunogenic\ mutations}{number\ of\ nonsilent\ mutations}$$

PyClone[23] v.0.13.1 was run to assess the clonality of predicted immunogenic mutations, defined as the proportion of tumour cells harbouring the mutation. PyClone was run independently for each region using tumour purity from the pathological assessment of slide A (Supplementary Table 2) and the gene copy number from ASCAT.

RNA sequencing

Total RNA was extracted from 58 macro-dissected regions of slides G1-5 in samples UH1-UH16 and 30 regions of slides F1-5 in samples UH17-UH23 and UH25-

UH27 (Supplementary Table 2) using the High Pure FFPE RNA isolation kit (Roche). For UH4, the two high and low CD3$^+$ regions were merged to obtain enough RNA for library preparation. RNA libraries were prepared starting from 5-50 ng of RNA using the QuantSeq 3'mRNA-seq Library Prep kit FWD for Illumina (Lexogen) and sequenced on Illumina HiSeq 4000 using 75 or 100 bp single end reads, according to manufacturer's instructions. Approximately 5-40 million reads were generated per sample.

Raw reads were processed using the Lexogen QuantSeq 3' mRNA-seq pipeline with default parameters[24]. Reads were first trimmed to remove Illumina adapters and polyA tails using bbduk from BBMap[25] v36.20. Trimmed reads were then aligned to GRCh38 reference human genome using STAR[26] v2.5.2a. Between 50-98% of the initial reads were retained after alignment and quality check. Gene expression was quantified using HTSeq[27] v0.6.1p1 and the GDC h38 GENCODE v22 GTF annotation file. To account for differences in sequencing depth across regions raw counts were normalised to the counts-per-million (CPM) gene expression unit calculated as:

$$CPM_{ij} = \frac{RC_{ij}}{\sum_k RC_{ik}} \times 10^6$$

where RC_{ij} is the raw read count of gene (j) in region (i). Since for samples UH1-UH16 RNA-seq was performed in six batches, potential batch effect was corrected using removeBatchEffect function from the Limma package[28] v3.36.5 on the log2-transformed CPM matrix with default parameters.

Differential gene expression between tumour groups was assessed using DESeq2[29] v1.20.0 from the raw read counts with default parameters with alpha set to 5%. To account for the experimental and clinical variability across samples (Supplementary Table 1), uncorrelated batch and clinical co-variates were included in the analysis (discovery cohort: batch effect, prior lines of treatment and Lynch syndrome in the comparison of DB- and nDB-CRCs of the; batch effect in the comparison of hypermutated and non-hypermutated CRCs. Validation cohort: prior lines of treatment, Lynch syndrome, treatment type and TNM staging).

A gene was considered as differentially expressed if DESEq2 Wald test FDR was <5% and had a fold change |>2|. Differentially expressed genes were used for pathway enrichment analysis in the three comparisons using MetaCore v20.3 build 70200 (Clarivate Analytics). Pathway enrichment was assessed through over-

representation analysis based on a hypergeometric test. A pathway was considered enriched if the FDR was <10%.

TCR sequencing

TCR-seq was performed in 28 macro-dissected regions in slides I1-5 of the discovery cohort, after excluding UH1, UH4 and UH5 because DNA was not sufficient (Supplementary Table 2). For UH12 all four regions were sequenced while for UH16 the two high and low $CD3^+$ regions were merged. For all remaining samples, the two regions corresponding to those used for IMC and WES were used.

DNA was extracted from macro-dissected regions (Supplementary Figure 1G) using GeneRead DNA FFPE kit (Qiagen) and submitted to Adaptive Biotechnologies (Seattle, USA) for non-lymphoid tissue (survey level) TCR-seq using a two-step, amplification bias-controlled multiplex PCR approach[30]. In the first step, V and J gene segments encoding the TCR beta CDR3 locus were amplified using reference gene primers to quantify total nucleated cells and measure the fraction of T cells in each sample. In the second step, proprietary barcodes and Illumina adapters were added. Finally, CDR3 and reference gene libraries were sequenced according to the manufacturer's instructions.

Raw reads were de-multiplexed and processed to remove adapter and primer sequences, identify and remove primer dimer, germline and other contaminant sequences. Resulting reads were clustered using both the relative frequency ratio between similar clones and a modified nearest-neighbour algorithm, to merge closely related sequences to correct for technical errors introduced through PCR and sequencing. The resulting reads were sufficient for annotating the V(N)D(N)J genes of each unique CDR3 and the translation of the encoded CDR3 amino acid sequence. V, D and J gene definitions were based on annotation in accordance with the IMGT database (www.imgt.org). The set of observed biological TCR Beta CDR3 sequences were normalised to correct for residual multiplex PCR amplification bias and quantified against a set of synthetic TCR Beta CDR3 sequence analogues[30]. Data was analysed using the immunoSEQ Analyzer toolset.

Detection of PD1-PDL1 interaction *in situ*

A total of 58 regions in slides K1-2 of the discovery cohort (Supplementary Table 2) were submitted to FASTBASE Solutions (Derio, Spain) to measure the interaction between PD1 and PDL1 *in situ* via amplified Förster Resonance Energy Transfer (A-FRET)[31]. Slides K1 were incubated overnight at 4 ºC with anti PD1 primary antibody (Supplementary Table 5) for donor only analyses. Slides K2 were stained with both anti PD1 and anti PDL1 primary antibodies for donor and acceptor analyses. Slides K1 were subsequently incubated with anti-mouse Fab-ATTO488 and slides K2 with both anti-mouse Fab-ATTO488 and anti-rabbit Fab-HRP. Alexa594 conjugated tyramide was added to slides K1 and K2 at 1/100 dilution in presence of 0,15% H_2O_2 and incubated at room temperature in the dark for 20 minutes. After washing in PBS and PBST twice, slides were mounted using Prolong Diamond Antifade Mount (Thermo Fisher), sealed and incubated at room temperature overnight before being transferred to a 4 ºC refrigerator for storage. FASTBASE Solutions SL frequency domain FLIM automated software programme was used to measure the excited-state lifetime of donor fluorophore (ATTO488) in both K1 and K2 slides. FRET efficiency E.was calculated as:

$$E = [1 - (tDA/tD)\, x100]$$

where *tDA* is donor lifetime in Slide K1 and *tD* is donor lifetime in Slide K2. *tDA* and *tD* values were collected for 793 optical fields of view (FOVs, with a median of 12 FOVs per region) in total to cover the whole surface of the regions analysed. Data were then collected in .csv files and imported into a macro spreadsheet programmed to calculate the A-FRET efficiency in each FOV. The results were finally expressed as the median FOV values per region.

Supplementary Figure 1. Experimental workflow, region selection and CD3 quantification

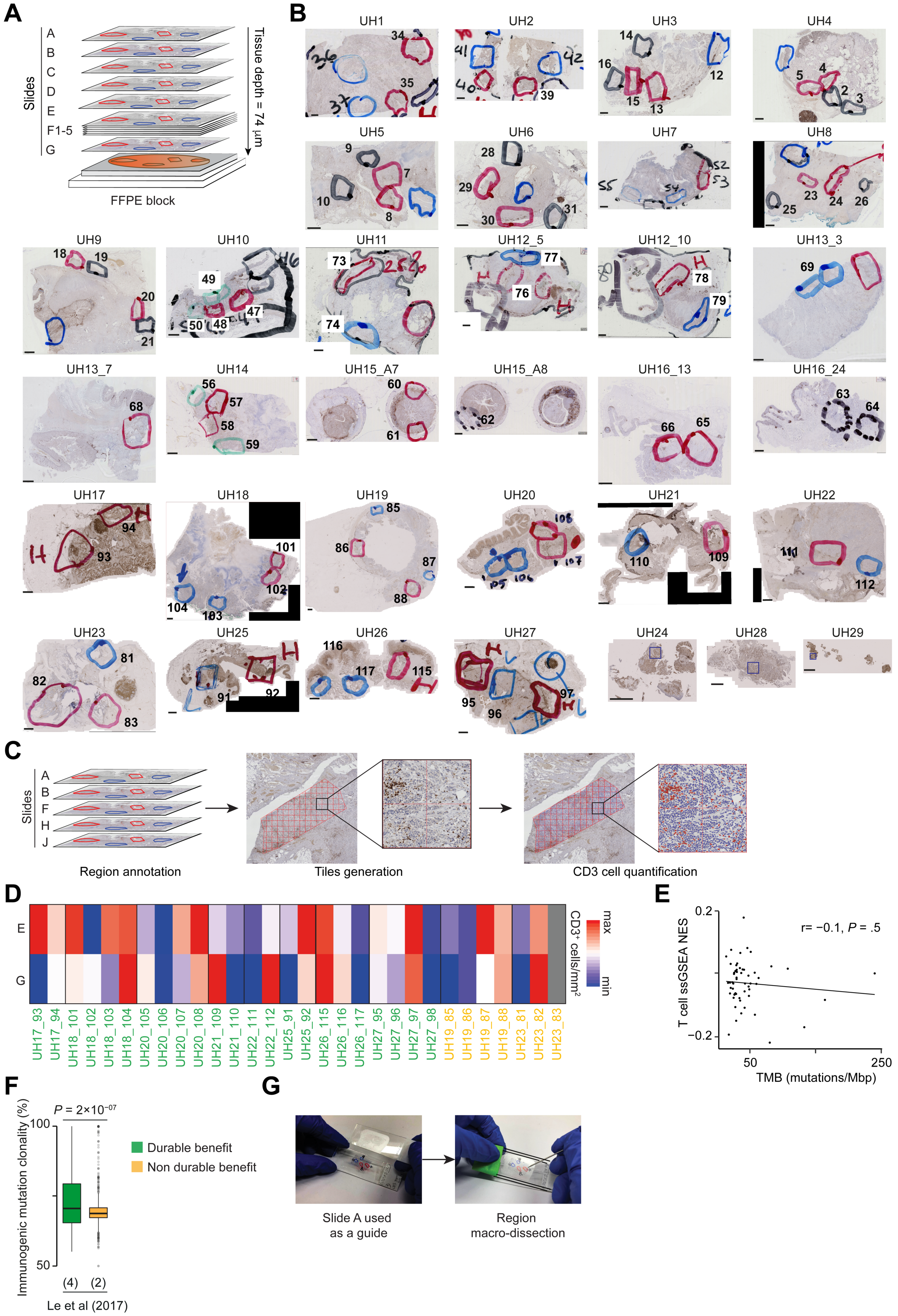

A. Experimental workflow for the analysis of the validation cohort. Eleven sequential sections from FFPE blocks of UH17-UH23 and UH25-UH27 were used for multiregional CD3 IHC (slides A, E and G), IMC with panel II (Table S5, slide C), mIF (slide D) and RNA-seq (slides F1-5). For the three biopsies (UH24, UH28, UH29) only IHC, IMC and mIF were performed.

B. Selected regions on slide A**.** Starting from slide A, CD3 staining was used by a board-certified pathologist (M.J.S.) to select multiple regions per sample with variable CD3 infiltrates and at the invasive margins of the tumour. For UH12, UH13, UH15, UH16 two different blocks were used. For biopsies (UH24, UH28 and UH29) shown are the IMC-ablated regions. Scale bar = 2mm.

C. Schematic of CD3 quantification. All slides immune-stained with anti-CD3 antibody were imported into QuPath[4]. Regions selected by the pathologist in slide A were projected into all other immune-stained slides and divided into 0.09 mm^2 tiles. $CD3^+$ cells were quantified within each tile and the CD3 content of a region was defined as the median number of $CD3^+$ cells per mm^2 across all tiles.

D. Quantification of $CD3^+$ cells/mm^2 from IHC staining in slides E and G in 30 regions of patients from the validation cohort using Qupath[4]. Values were normalised within each patient. Grey boxes indicate missing values.

E. Correlation between the T cell signature normalised enrichment scores (NES) from[32] and TMB in 56 hypermutated CRCs from TCGA. Pearson correlation coefficient and associated p-value are shown. ssGSEA, single sample gene set enrichment analysis.

F. Comparison of clonality of immunogenic mutations between four DB and two nDB-CRCs with purity >30% from[33]. For two samples (Subjects 33 and 36) the reference counts were randomly sampled from the rest of samples and the variant counts were subsequently calculated. Given the lack of copy number data, PyClone[23] was ran with minor_cn=0, major_cn=2, prior=total_copy_number. Due to lack of expression data, immunogenic mutations were considered as expressed if contained in 11,056 genes expressed in >30% of TCGA CRCs.

G. Macro-dissection for DNA and RNA extraction. Regions selected in slide A were used as a reference for the macro-dissection of all slides used for DNA and RNA extraction. Each slide was aligned to slide A using a stereo microscope and regions were manually dissected with a needle. The collected tissue was subsequently used for DNA or RNA extraction

Supplementary Figure 2. Genetic and TME features in DB and nDB-CRCs

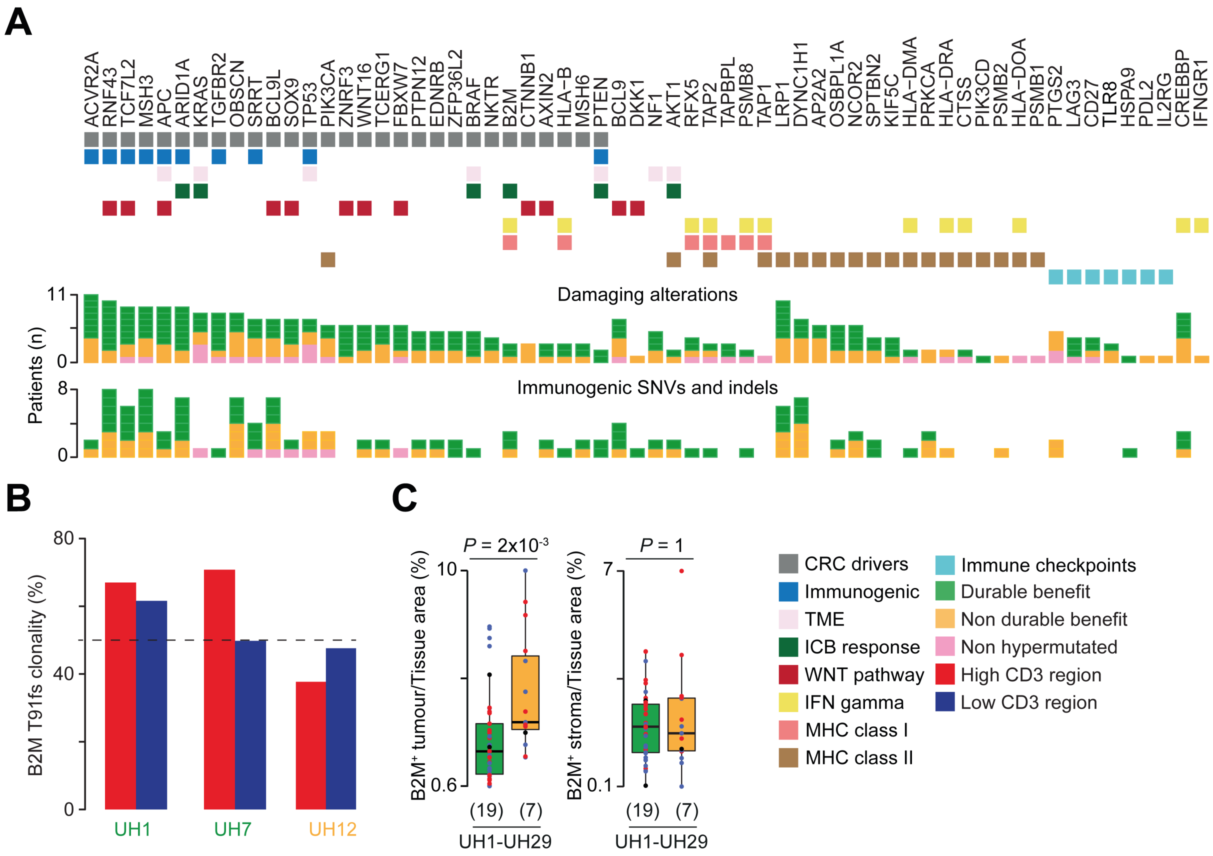

A. Predicted damaging alterations (truncating and missense damaging alterations, double hits, gene amplifications leading to increased expression, gene homozygous deletions) and immunogenic mutations of representative genes from a manually curated list of 647 genes including common CRC drivers[34]; genes whose alterations are immunogenic[35, 36]; genes that modify the TME[37-41], modulate the response to immune checkpoint inhibitors[38-40, 42-45], or encode members of WNT[41, 46] and IFN-gamma pathways (MetaCore Clarivate Analytics), components of the antigen presentation machinery via the major histocompatibility complex (MHC) class I[47, 48] or class II (MetaCore, Clarivate Analytics), and immune checkpoints[49, 50].

B. Clonality of *B2M* truncating mutation (T91fs) in all sequenced regions from two DB and one nDB-CRCs. Clonality was measured using Pyclone[23] after correction for purity and copy number alterations (Methods).

C. Comparison of tumour and stroma B2M⁺ areas between DB- and nDB-CRCs in all analysed samples. Distributions were compared using two-sided Wilcoxon rank sum test and the number of patients in each group is reported in brackets.

TME, tumour microenvironment; ICB, Immune Checkpoint Blockade; IFN, Interferon; MHC, Major Histocompatibility Complex; IC, Immune Checkpoints.

Supplementary Figure 3. Proportion of tumour and stroma from IMC across samples

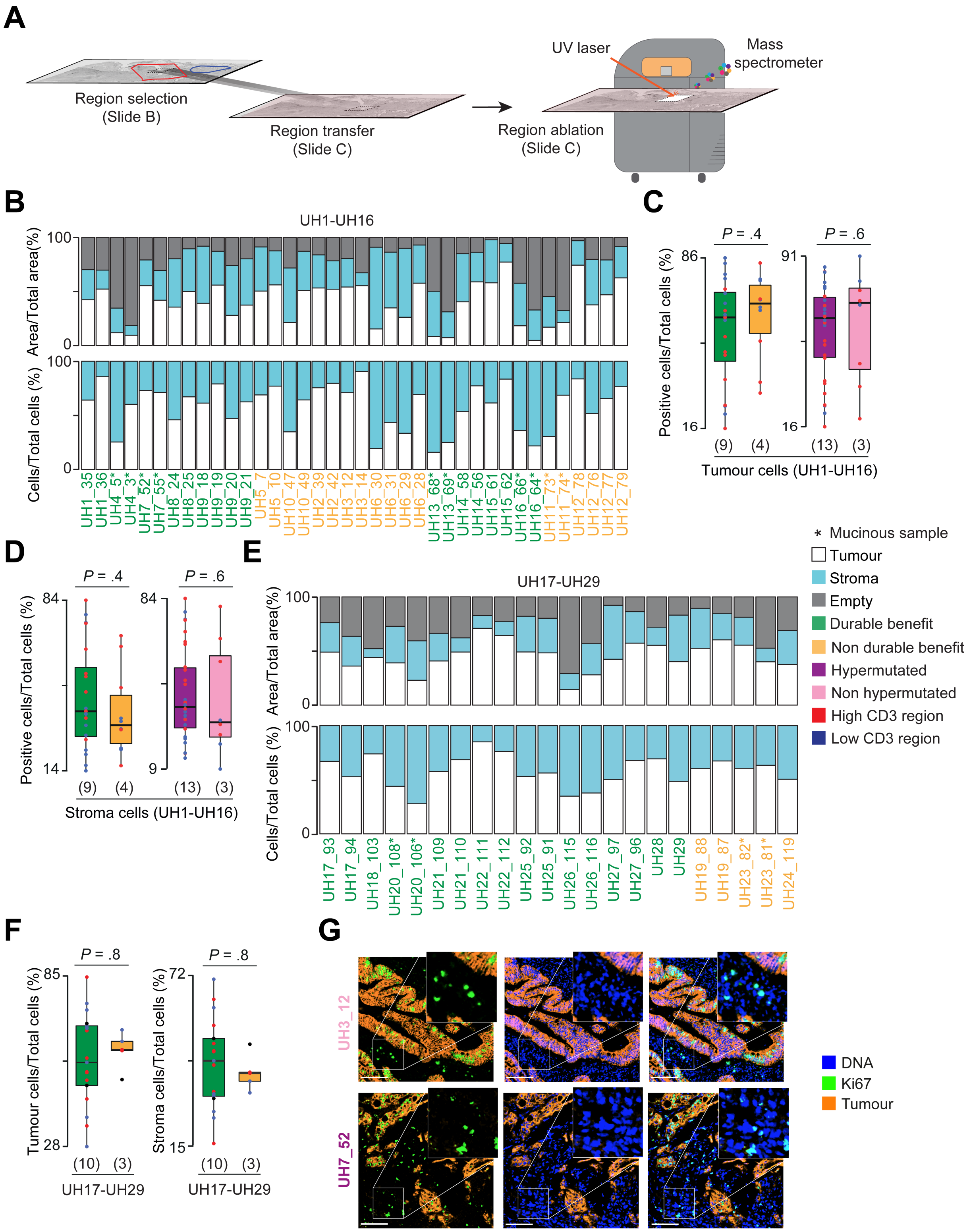

A. IMC experimental workflow. Representative 1mm^2 areas in slide B were projected into slide C using the macroscopic tissue structure as a reference. Slide D stained with the IMC antibody panel was loaded in the Hyperion Imaging System (Fluidigm) for regional ablation.

B. Proportions of tumour areas and cells in ablated regions of the discovery cohort. Areas not covered by stroma or tumour are depicted in grey.

Comparison of the proportion of tumour **(C)** and stroma cells **(D)** over total cells between DB and nDB-CRCs or hypermutated and non-hypermutated CRCs in the discovery cohort.

E. Proportions of tumour areas and cells in ablated regions of the validation cohort. Areas not covered by stroma or tumour are depicted in grey.

F. Comparison of the proportion of tumour and stroma cells over total cells between DB and nDB-CRCs in the validation cohort.

All distributions were compared using two-sided Wilcoxon rank sum test and the number of patients in each group is reported in brackets.

G. IMC-derived images of tumour-associated markers (E-cadherin and Pan-Keratin), Ki67 and DNA staining in two representative hypermutated and non-hypermutated CRCs. Scale bar = 100μm.

Supplementary Figure 4. Protein expression heatmaps from single cell clustering

A

DB- vs nDB-CRCs UH1-UH16 (13 samples, 30 regions)

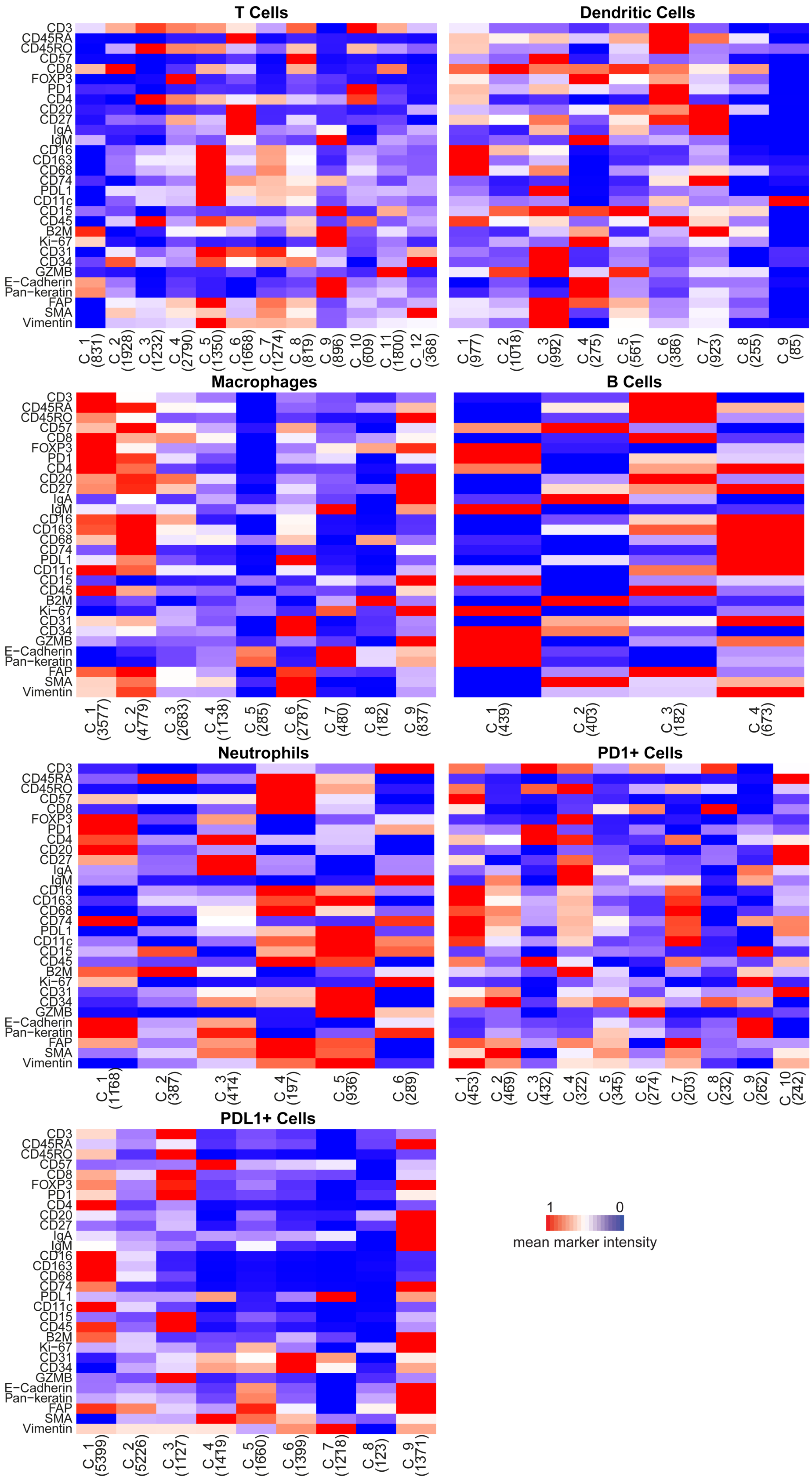

T cells

C_1 CD3, CD8, Ki−67
C_2 CD3, CD8
C_3 CD3, CD45RO, CD4
C_4 CD3, CD45RO, FOXP3, CD4
C_5 CD3, CD45RO, CD8, CD4, CD16
C_6 CD3, CD45RA, CD8, CD4
C_7 CD4
C_8 CD3, CD45RO, CD57, CD8
C_9 E−Cadherin, Ki−67
C_10 CD3, CD45RO, PD1, CD4
C_11 CD3, CD8, GZMB
C_12 CD3

Dendritic Cells

C_1 CD16
C_2 CD16, CD11c
C_3 Vimentin
C_4 Pan−keratin, E−Cadherin, Ki−67
C_5 Pan−keratin, E−Cadherin, CD11c
C_6 CD4, CD11c
C_7 CD74
C_8 CD16, CD163, CD11c
C_9 CD11c

Macrophages

C_1 CD4, CD16, CD163, CD68
C_2 Vimentin, CD16, CD163, CD68, CD74
C_3 CD16, CD68
C_4 CD68
C_5 Pan−keratin, E−Cadherin, CD68
C_6 Vimentin, CD68
C_7 Pan−keratin, E−Cadherin
C_8 Pan−keratin, CD68
C_9 Pan−keratin

B Cells

C_1 Pan−keratin, E−Cadherin, Ki−67
C_2 CD45RA
C_3 CD45RA, CD20, CD27
C_4 CD45RA, CD27

Neutrophils

C_1 Pan−keratin, E−Cadherin, CD15, Ki−67
C_2 CD15
C_3 Pan−keratin, E−Cadherin, CD15
C_4 CD45RO, CD15
C_5 CD15, GZMB
C_6 Pan−keratin, CD15, Ki−67, GZMB

PD1+ Cells

C_1 CD3, CD45RO, CD57, CD8, CD4, CD16
C_2 CD3, CD4
C_3 CD3, CD45RO, PD1, CD4
C_4 CD3, CD45RO, FOXP3, CD4
C_5 CD3, CD8
C_6 CD3, CD45RO, CD8, GZMB
C_7 CD3, CD45RO, CD4, CD16
C_8 CD3, CD45RO, CD8
C_9 CD4, E−Cadherin, Ki−67
C_10 CD3, CD45RA, CD45RO, CD4

PDL1+ Cells

C_1 Vimentin, CD16, CD163, CD68, CD74, PDL1
C_2 FAP, SMA, Vimentin, CD16, PDL1
C_3 CD45RO, SMA, Vimentin, PDL1
C_4 SMA, Vimentin, PDL1
C_5 SMA
C_6 SMA, Vimentin, CD31
C_7 Vimentin, PDL1, CD31
C_8 FAP
C_9 Vimentin, CD74, PDL1

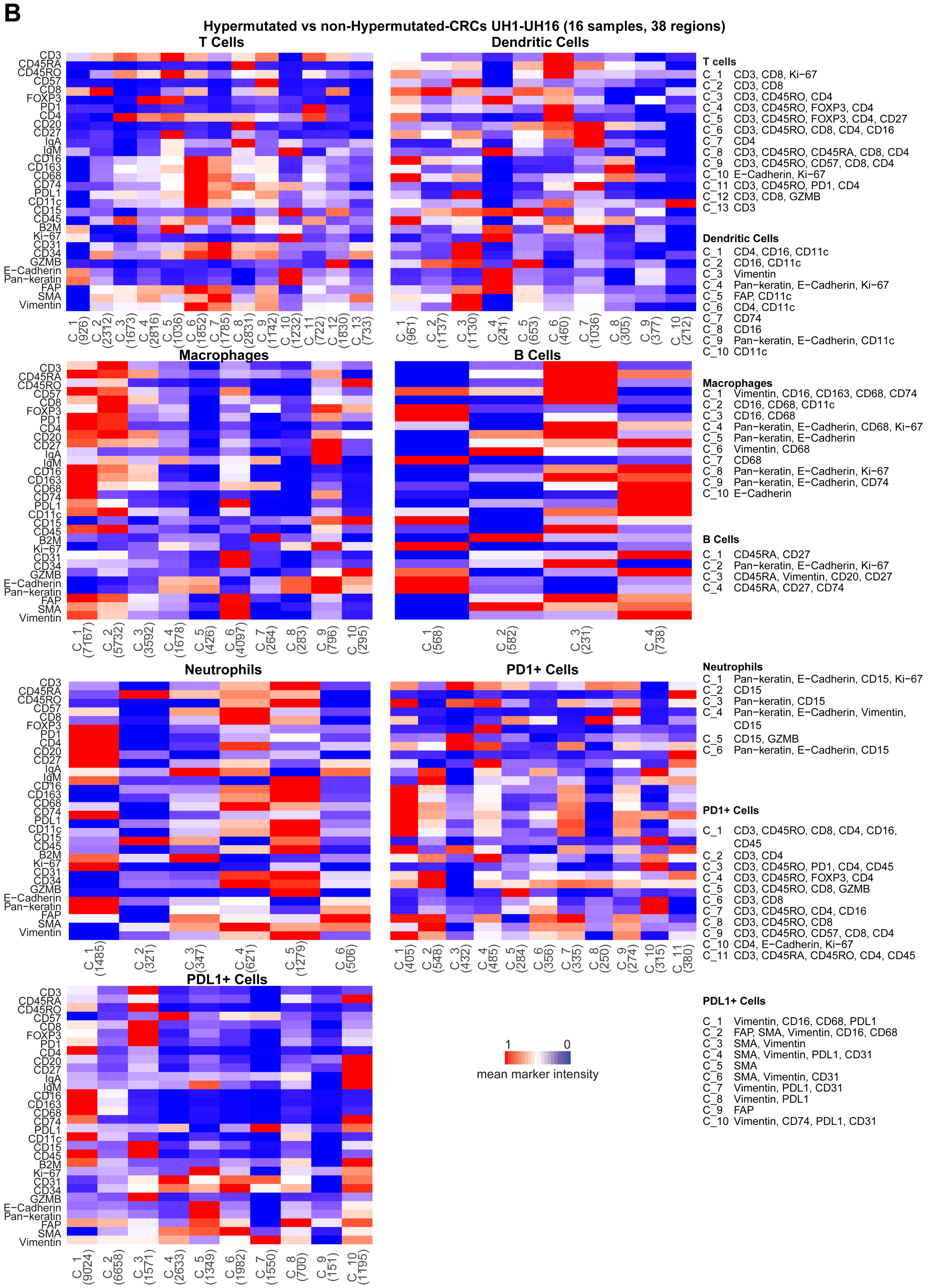
B
Hypermutated vs non-Hypermutated-CRCs UH1-UH16 (16 samples, 38 regions)
T Cells
Dendritic Cells
Macrophages
B Cells
Neutrophils
PD1+ Cells
PDL1+ Cells
mean marker intensity
T cells
C_1 CD3, CD8, Ki−67
C_2 CD3, CD8
C_3 CD3, CD45RO, CD4
C_4 CD3, CD45RO, FOXP3, CD4
C_5 CD3, CD45RO, FOXP3, CD4, CD27
C_6 CD3, CD45RO, CD8, CD4, CD16
C_7 CD4
C_8 CD3, CD45RO, CD45RA, CD8, CD4
C_9 CD3, CD45RO, CD57, CD8, CD4
C_10 E−Cadherin, Ki−67
C_11 CD3, CD45RO, PD1, CD4
C_12 CD3, CD8, GZMB
C_13 CD3
Dendritic Cells
C_1 CD4, CD16, CD11c
C_2 CD16, CD11c
C_3 Vimentin
C_4 Pan−keratin, E−Cadherin, Ki−67
C_5 FAP, CD11c
C_6 CD4, CD11c
C_7 CD74
C_8 CD16
C_9 Pan−keratin, E−Cadherin, CD11c
C_10 CD11c
Macrophages
C_1 Vimentin, CD16, CD163, CD68, CD74
C_2 CD16, CD68, CD11c
C_3 CD16, CD68
C_4 Pan−keratin, E−Cadherin, CD68, Ki−67
C_5 Pan−keratin, E−Cadherin
C_6 Vimentin, CD68
C_7 CD68
C_8 Pan−keratin, E−Cadherin, Ki−67
C_9 Pan−keratin, E−Cadherin, CD74
C_10 E−Cadherin
B Cells
C_1 CD45RA, CD27
C_2 Pan−keratin, E−Cadherin, Ki−67
C_3 CD45RA, Vimentin, CD20, CD27
C_4 CD45RA, CD27, CD74
Neutrophils
C_1 Pan−keratin, E−Cadherin, CD15, Ki−67
C_2 CD15
C_3 Pan−keratin, CD15
C_4 Pan−keratin, E−Cadherin, Vimentin, CD15
C_5 CD15, GZMB
C_6 Pan−keratin, E−Cadherin, CD15
PD1+ Cells
C_1 CD3, CD45RO, CD8, CD4, CD16, CD45
C_2 CD3, CD4
C_3 CD3, CD45RO, PD1, CD4, CD45
C_4 CD3, CD45RO, FOXP3, CD4
C_5 CD3, CD45RO, CD8, GZMB
C_6 CD3, CD8
C_7 CD3, CD45RO, CD4, CD16
C_8 CD3, CD45RO, CD8
C_9 CD3, CD45RO, CD57, CD8, CD4
C_10 CD4, E−Cadherin, Ki−67
C_11 CD3, CD45RA, CD45RO, CD4, CD45
PDL1+ Cells
C_1 Vimentin, CD16, CD68, PDL1
C_2 FAP, SMA, Vimentin, CD16, CD68
C_3 SMA, Vimentin
C_4 SMA, Vimentin, PDL1, CD31
C_5 SMA
C_6 SMA, Vimentin, CD31
C_7 Vimentin, PDL1, CD31
C_8 Vimentin, PDL1
C_9 FAP
C_10 Vimentin, CD74, PDL1, CD31

C

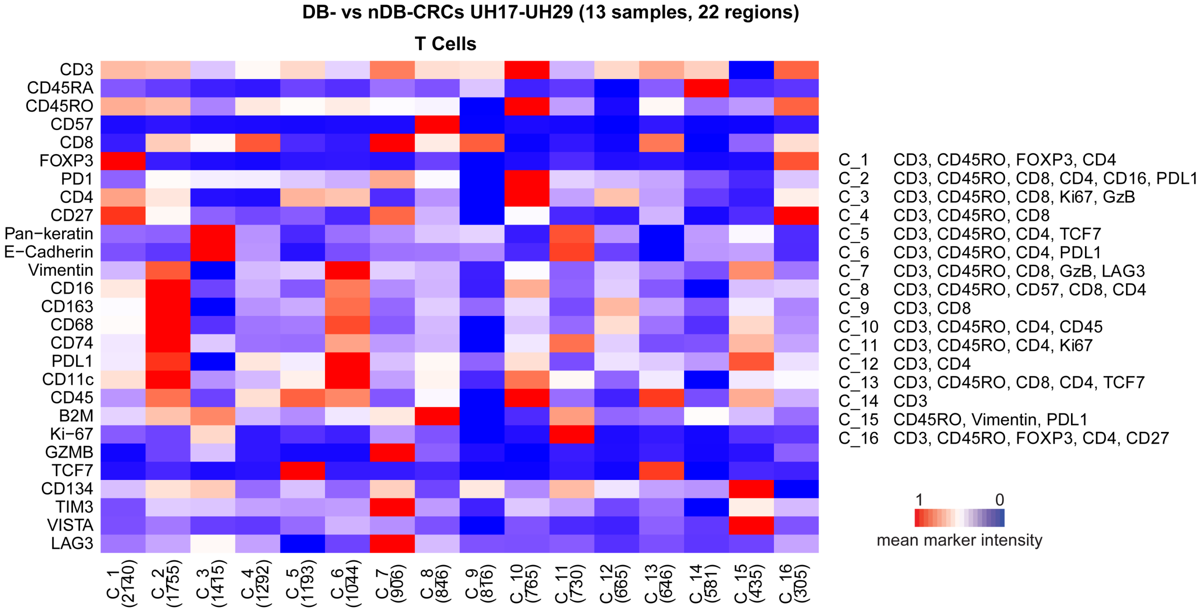

Subpopulations (clusters) of T cells, dendritic cells, macrophages, B cells, neutrophils, PD1⁺ cells, and PDL1⁺ cells were identified based on the expression of phenotypic markers using Seurat[9] in the discovery cohort. For each subpopulation, the mean value of 30 markers of IMC Panel I (Table S5) across the cells in that cluster is reported. For each cluster, the number of cells is reported in brackets. Single cell clustering was performed separately for DB vs nDB-CRCs **(A)** and hypermutated vs non-hypermutated CRCs **(B)**.

C. T cell subpopulations in the validation cohort. In this case, T cells were clustered using 17 markers and the mean value of 27 markers of IMC Panel II (Table S5) across the cells in that cluster is reported.

For each cell population the colour scale was normalized separately for each marker across all analysed clusters.

Supplementary Figure 5. CD74^{+} macrophage identification and phenotyping

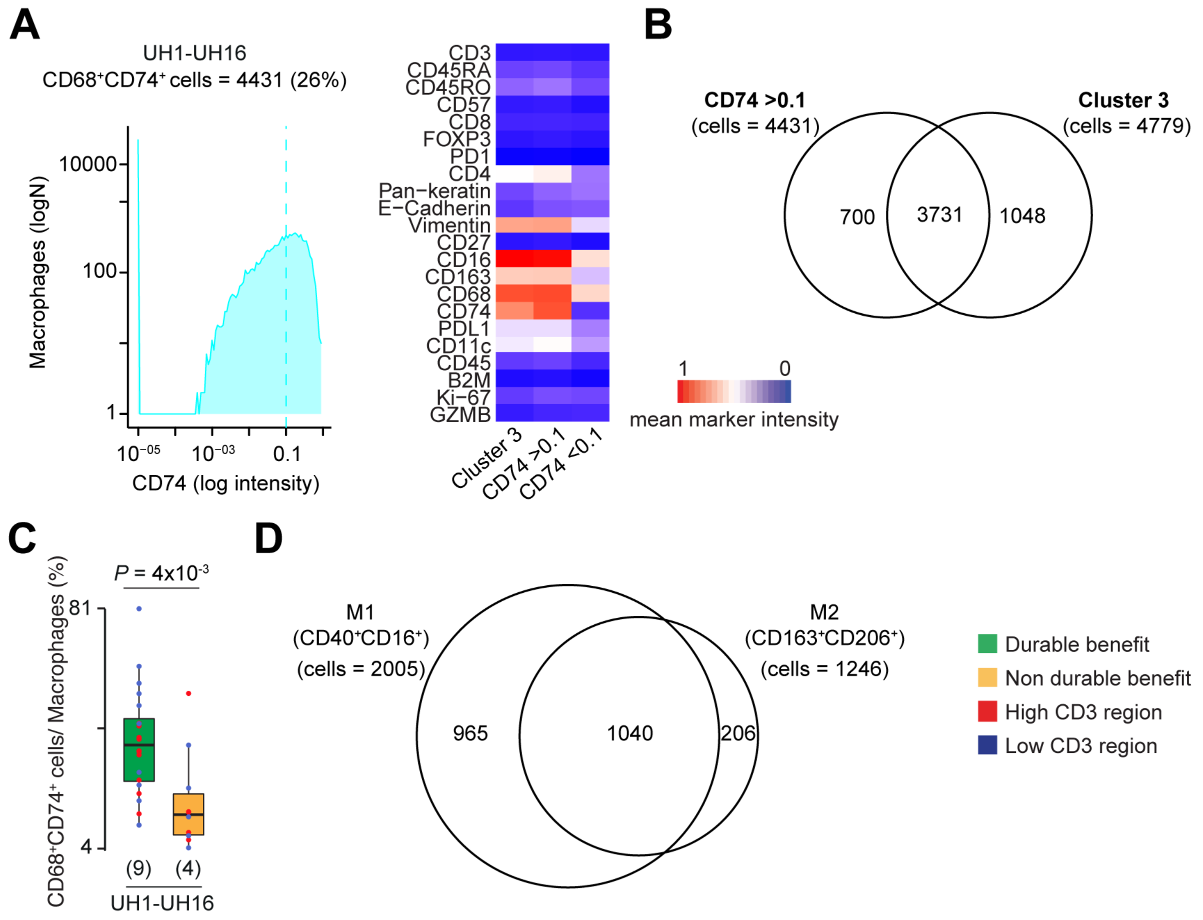

A. CD74^{+} macrophages in the discovery cohort identified by applying 0.1 threshold on CD74 expression. The mean intensities of IMC markers in CD74^{+} and CD74^{-} macrophages are reported. Colour gradient was normalised across all markers and cells.

B. Overlap between CD74^{+} macrophages identified with 0.1 CD74 expression and cluster 3 in the discovery cohort.

C. Comparison of CD74^{+} macrophages between DB- and nDB-CRCs in the discovery cohort. CD74^{+} macrophages were identified by applying a threshold of 0.1 CD74 expression to all macrophages.

D. Overlap between CD74^{+} macrophages expressing M1 and M2-associated markers. Used thresholds after histological inspection of IMC images were: 0.2 for CD40; 0.1 for CD16; 0.15 for CD163 and 0.1 for CD206.

All distributions were compared using two-sided Wilcoxon rank sum test and the number of patients in each group is reported in brackets

Supplementary Figure 6. Comparison of PD1 and PDL1 gene and protein expression

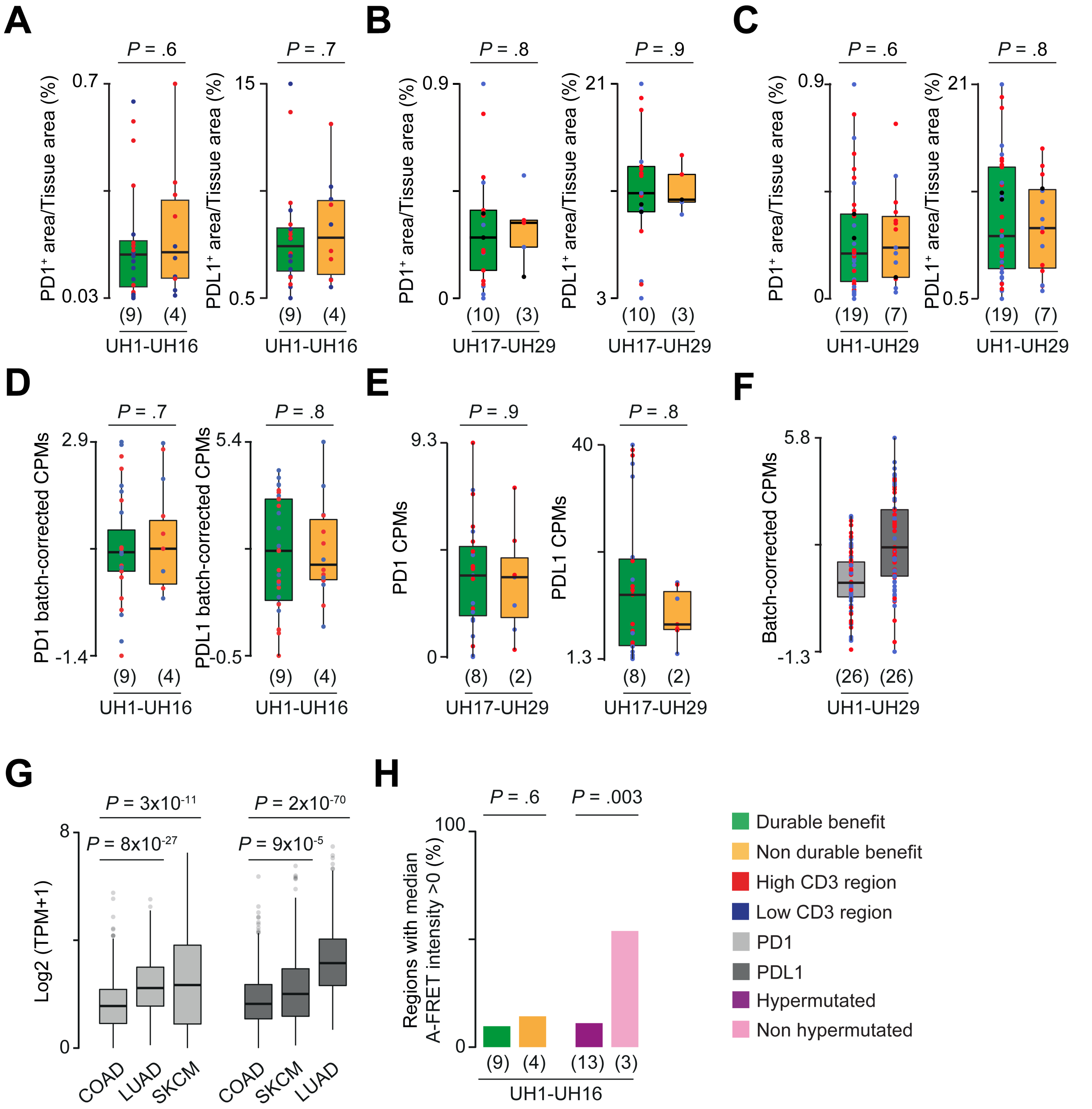

Comparison of normalised PD1⁺ and PDL1⁺ areas between DB and nDB-CRCs from the discovery **(A)** and validation **(B)** cohorts.

C. Comparison of normalised PD1⁺ and PDL1⁺ positive areas in all samples analysed.

Comparison of *PD1* and *PDL1* Counts Per Million (CPMs) expression levels in the discovery **(D)** and validation **(E)** cohorts. For the discovery samples batch correction was applied (Methods).

F. *PD1* and *PDL1* gene expression in all regions from all samples analysed. CPMs were obtained from RNAseq raw counts and corrected for batch effects.

G. Comparison of *PD1* and *PDL1* gene expression in colon adenocarcinoma (COAD), lung adenocarcinoma (LUAD), and skin cutaneous melanoma (SKCM) from TCGA. Transcripts Per Million (TPM) were computed from raw read counts.

H. Percentage of regions with median A-FRET intensity higher than zero in DB and nDB-CRCs and hypermutated and non-hypermutated CRCs. Proportions were compared using Fisher's exact test.

All distributions were compared using two-sided Wilcoxon rank sum test and the number of patients in each group is reported in brackets.

Supplementary Figure 7. Proliferating and cytotoxic CD8 T cell identification

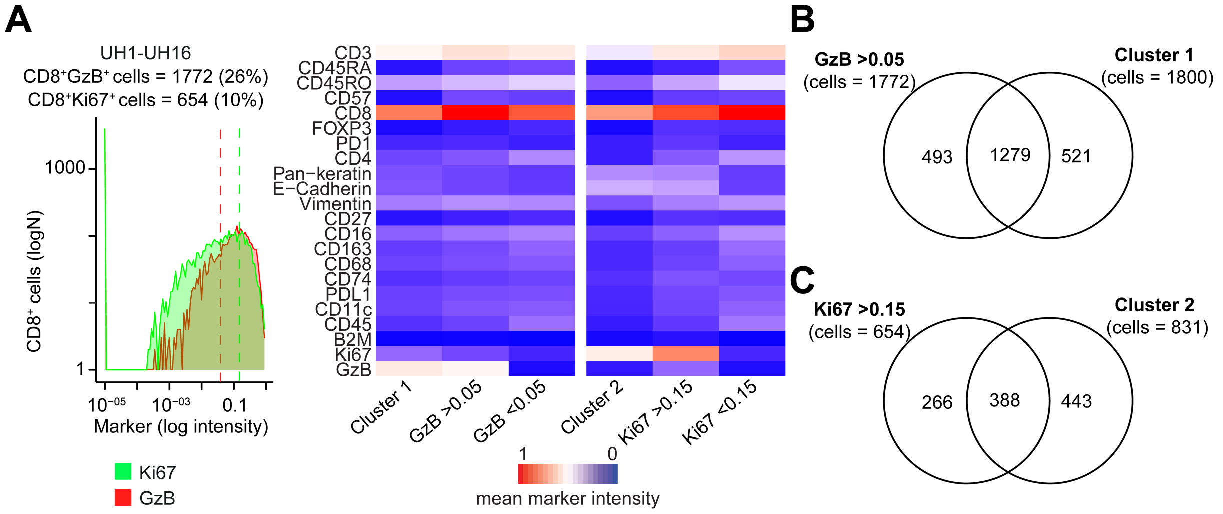

A. $CD8^{+}GzB^{+}$ and $CD8^{+}Ki67^{+}$ cells in the discovery cohort identified by applying a threshold of 0.05 GzB and 0.15 Ki67 expression to CD8 T cells, respectively. The mean intensities of IMC markers in $CD8^{+}GzB^{+}$ or $CD8^{+}Ki67^{+}$ and $CD8^{+}GzB^{-}$ or $CD8^{+}Ki67^{-}$ T cells are reported. Colour scale was normalised across all markers and cells.

Overlap between $CD8^{+}GzB^{+}$ cells and cluster 1 **(B)** or $CD8^{+}Ki67^{+}$ cells and cluster 2 **(C)** cells in the discovery cohort. Positive cells were identified using and expression threshold as described in **(A)**.

Supplementary Figure 8. High-density CD74$^+$ macrophage clusters

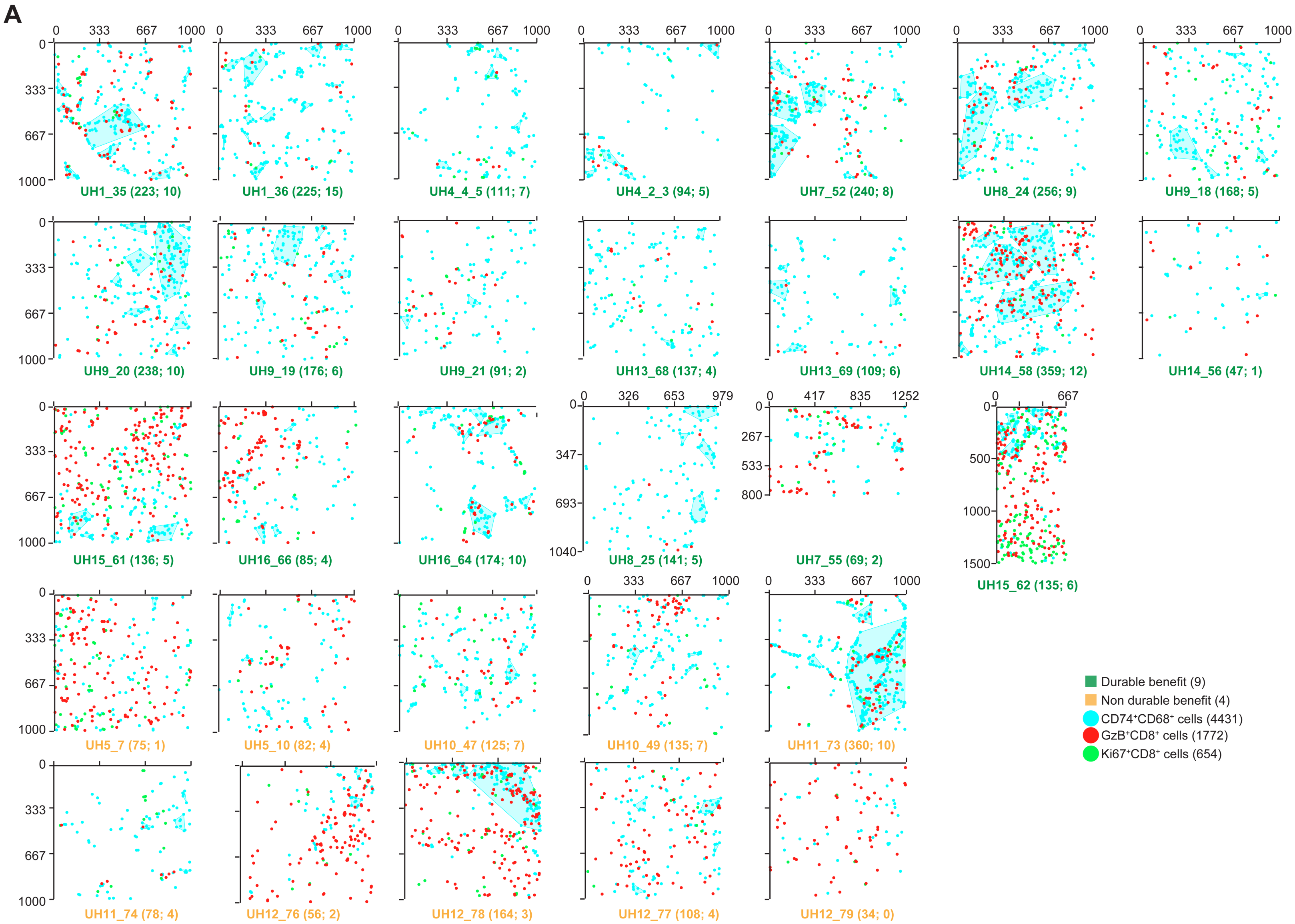

B

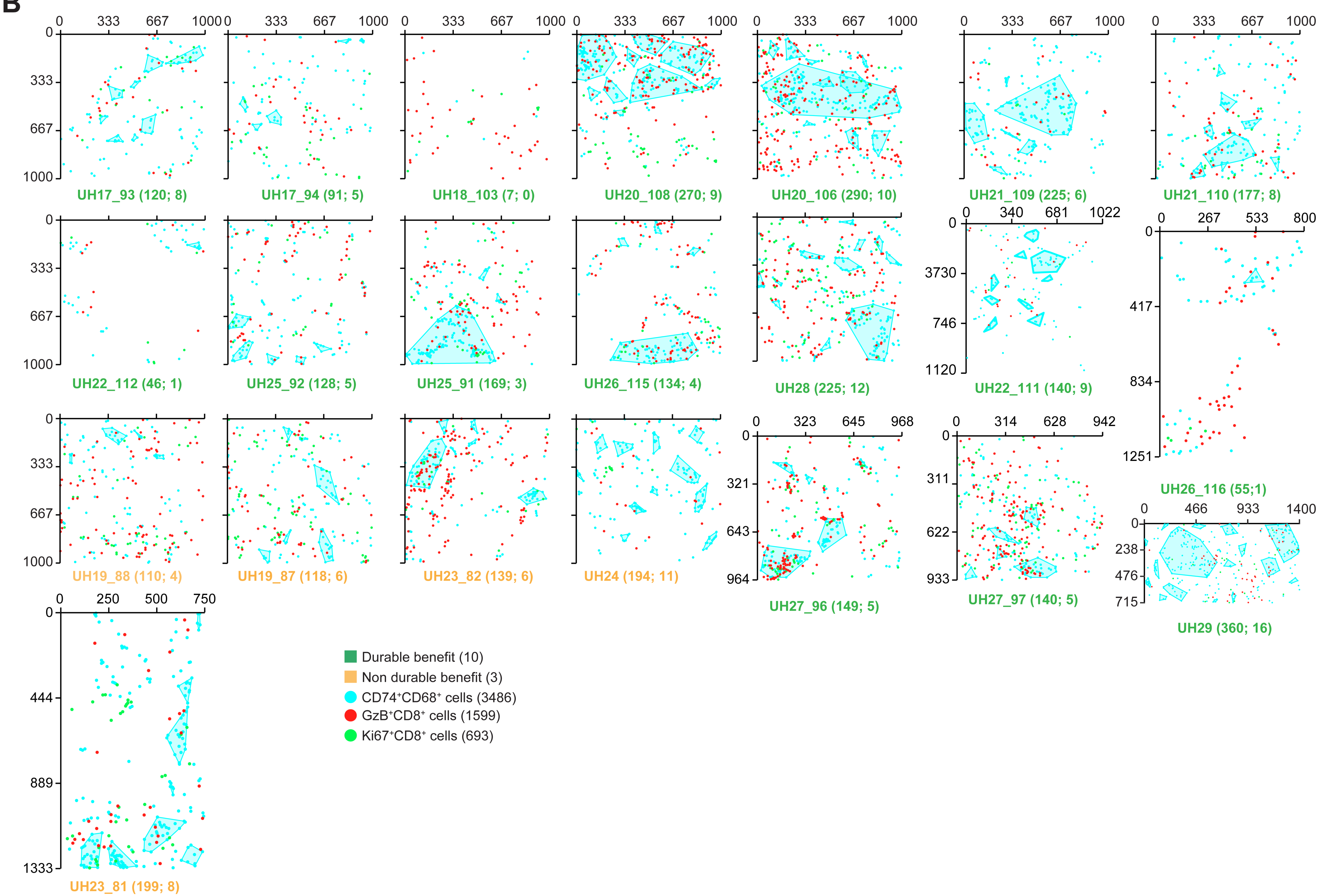

UH17_93 (120; 8)
UH17_94 (91; 5)
UH18_103 (7; 0)
UH20_108 (270; 9)
UH20_106 (290; 10)
UH21_109 (225; 6)
UH21_110 (177; 8)
UH22_112 (46; 1)
UH25_92 (128; 5)
UH25_91 (169; 3)
UH26_115 (134; 4)
UH28 (225; 12)
UH22_111 (140; 9)
UH26_116 (55;1)
UH19_88 (110; 4)
UH19_87 (118; 6)
UH23_82 (139; 6)
UH24 (194; 11)
UH27_96 (149; 5)
UH27_97 (140; 5)
UH29 (360; 16)
UH23_81 (199; 8)
Durable benefit (10)
Non durable benefit (3)
CD74+CD68+ cells (3486)
GzB+CD8+ cells (1599)
Ki67+CD8+ cells (693)

High-density cluster maps of CD68^{+}CD74^{+} cells in 52 IMC regions from hypermutated CRCs of the discovery **(A)** and validation **(B)** cohorts. Clusters were identified from cell pixel coordinates as portions of the ablated region with ≥5 CD68^{+}/CD74^{+} cells per 10,000μm^2 (Methods). CD8^{+}/GzB^{+} and CD8^{+}/Ki67^{+} cells were subsequently mapped. The number of CD68^{+}/CD74^{+} cells and the number of high-density clusters are reported in brackets for each region.

Supplementary Figure 9. Examples of interactions between CD74^{+} macrophages and cytotoxic or proliferating CD8 T cells by IMC

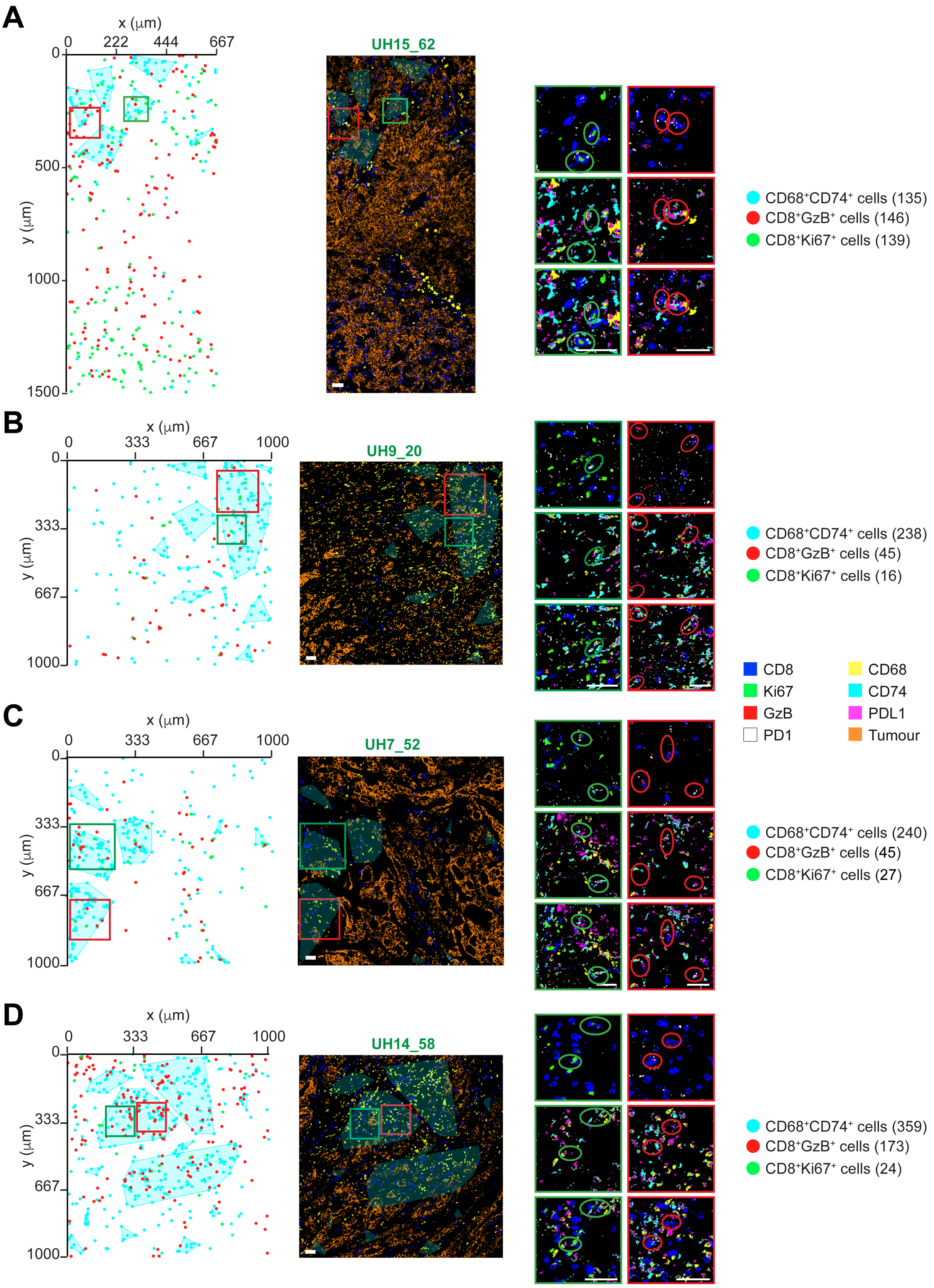

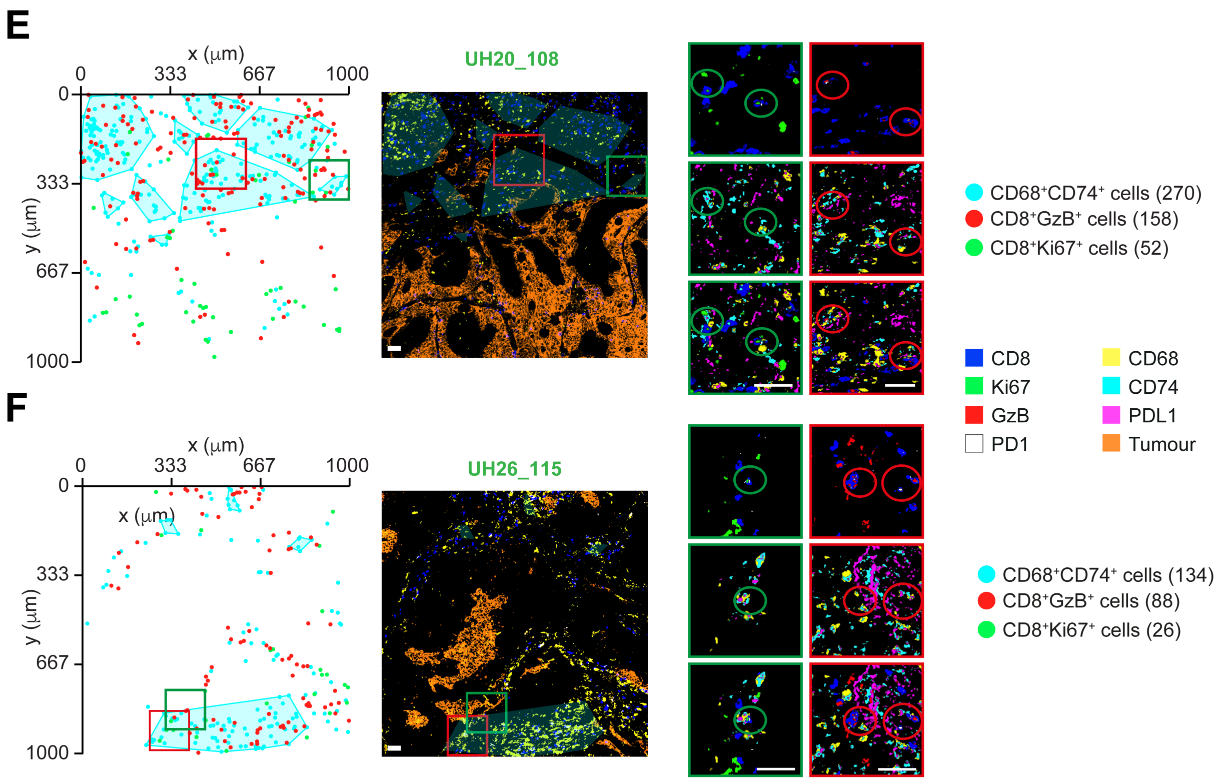

High-density cluster maps of CD68$^+$/CD74$^+$ cells in representative IMC regions from the discovery **(A-D)** and validation **(E,F)** cohorts. Clusters identified computationally (left panels) as described in Supplementary Figure 8 and in Methods. Red and green squares indicate areas of interest that were identified independently via histological inspection (middle panels). In these area CD68$^+$/CD74$^+$/PDL1$^+$ cells interact with CD8$^+$/Ki67$^+$/PD1$^+$ cells (green) and CD8$^+$/GzB$^+$/PD1$^+$ cells (red). These areas are further detailed (right panels) to show the cellular interactions between CD68$^+$/CD74$^+$/PDL1$^+$ cells and CD8$^+$/Ki67$^+$/PD1$^+$ cells (green circles) and CD8$^+$/GzB$^+$/PD1$^+$ cells (red circles). Images were compiled overlaying single-marker images obtained applying a median filter. For each region, number of cells are reported in brackets. Scale bar = 50μm.

Supplementary Figure 10. Examples of interactions between CD74^{+} macrophages and cytotoxic or proliferating CD8 T cells by mIF

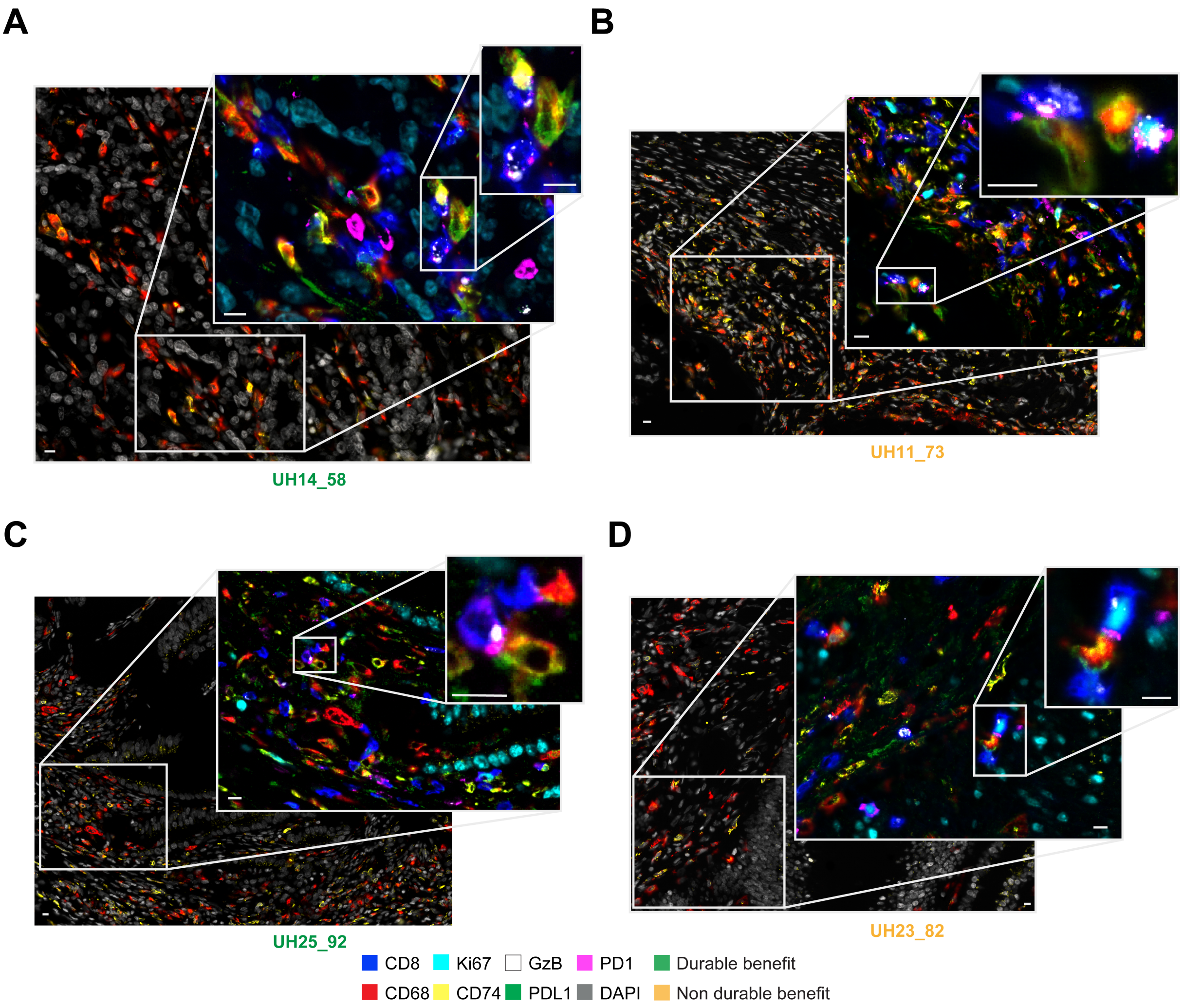

High resolution (40x) images of cellular interactions between CD68^{+}CD74^{+}PDL1^{+} cells and CD8^{+}PD1^{+}GzB^{+} and CD8^{+}PD1^{+}Ki67^{+}GzB^{+} cells within high-density clusters of CD68^{+}CD74^{+} cells in representative DB- and nDB-CRCs from the discovery **(A,B)** and validation **(C,D)** cohorts. Scale bar = 10 µm.

References

1. André T, Shiu K-K, Kim TW, et al. Pembrolizumab in Microsatellite-Instability–High Advanced Colorectal Cancer. New England Journal of Medicine 2020;383:2207-2218.
2. Hoos A, Eggermont AM, Janetzki S, et al. Improved endpoints for cancer immunotherapy trials. J Natl Cancer Inst 2010;102:1388-97.
3. Shi Q, de Gramont A, Grothey A, et al. Individual patient data analysis of progression-free survival versus overall survival as a first-line end point for metastatic colorectal cancer in modern randomized trials: findings from the analysis and research in cancers of the digestive system database. J Clin Oncol 2015;33:22-8.
4. Bankhead P, Loughrey MB, Fernandez JA, et al. QuPath: Open source software for digital pathology image analysis. Sci Rep 2017;7:16878.
5. Bortolomeazzi M, Montorsi L, Temelkovski D, et al. A SIMPLI (Single-cell Identification from MultiPLexed Images) approach for spatially resolved tissue phenotyping at single-cell resolution. bioRxiv 2021:2021.04.01.437886.
6. imctools. https://github.com/BodenmillerGroup/imctools, 2018.
7. McQuin C, Goodman A, Chernyshev V, et al. CellProfiler 3.0: Next-generation image processing for biology. PLoS Biol 2018;16:e2005970.
8. Berg S, Kutra D, Kroeger T, et al. ilastik: interactive machine learning for (bio)image analysis. Nat Methods 2019;16:1226-1232.
9. Butler A, Hoffman P, Smibert P, et al. Integrating single-cell transcriptomic data across different conditions, technologies, and species. Nat Biotechnol 2018;36:411-420.
10. Ester M, Kriegel H-P, Sander J, et al. A density-based algorithm for discovering clusters in large spatial databases with noise. Proceedings of the Second International Conference on Knowledge Discovery and Data Mining 1996:226–231.
11. Li H. Aligning sequence reads, clone sequences and assembly contigs with BWA-MEM. Preprint at: https://arxiv.org/abs/1303.3997 2013.
12. McKenna A, Hanna M, Banks E, et al. The Genome Analysis Toolkit: a MapReduce framework for analyzing next-generation DNA sequencing data. Genome Res 2010;20:1297-303.
13. Kim S, Scheffler K, Halpern AL, et al. Strelka2: fast and accurate calling of germline and somatic variants. Nat Methods 2018;15:591-594.
14. Frampton GM, Fichtenholtz A, Otto GA, et al. Development and validation of a clinical cancer genomic profiling test based on massively parallel DNA sequencing. Nat Biotechnol 2013;31:1023-31.
15. Van Loo P, Nordgard SH, Lingjaerde OC, et al. Allele-specific copy number analysis of tumors. Proc Natl Acad Sci U S A 2010;107:16910-5.
16. Castel SE, Levy-Moonshine A, Mohammadi P, et al. Tools and best practices for data processing in allelic expression analysis. Genome Biol 2015;16:195.
17. Consortium GP. A global reference for human genetic variation. Nature 2015;526:68-74.
18. Wang K, Li M, Hakonarson H. ANNOVAR: functional annotation of genetic variants from high-throughput sequencing data. Nucleic Acids Res 2010;38:e164.
19. Mourikis TP, Benedetti L, Foxall E, et al. Patient-specific cancer genes contribute to recurrently perturbed pathways and establish therapeutic vulnerabilities in esophageal adenocarcinoma. Nat Commun 2019;10:3101.
20. Tamborero D, Gonzalez-Perez A, Lopez-Bigas N. OncodriveCLUST: exploiting the positional clustering of somatic mutations to identify cancer genes. Bioinformatics 2013;29:2238-44.

21. Shukla SA, Rooney MS, Rajasagi M, et al. Comprehensive analysis of cancer-associated somatic mutations in class I HLA genes. Nat Biotechnol 2015;33:1152-8.

22. Schenck RO, Lakatos E, Gatenbee C, et al. NeoPredPipe: high-throughput neoantigen prediction and recognition potential pipeline. BMC Bioinform 2019;20:264.

23. Roth A, Khattra J, Yap D, et al. PyClone: statistical inference of clonal population structure in cancer. Nat methods 2014;11:396-398.

24. Moll P, Ante M, Seitz A, et al. QuantSeq 3′ mRNA sequencing for RNA quantification. Nat Methods 2014;11:i-iii.

25. Bushnell B. BBMap: a fast, accurate, splice-aware aligner. https://www.osti.gov/biblio/1241166 , 2014.

26. Dobin A, Davis CA, Schlesinger F, et al. STAR: ultrafast universal RNA-seq aligner. Bioinformatics 2013;29:15-21.

27. Anders S, Pyl PT, Huber W. HTSeq—a Python framework to work with high-throughput sequencing data. Bioinformatics 2015;31:166-169.

28. Ritchie ME, Phipson B, Wu D, et al. limma powers differential expression analyses for RNA-sequencing and microarray studies. Nucleic Acids Res 2015;43:e47.

29. Love MI, Huber W, Anders S. Moderated estimation of fold change and dispersion for RNA-seq data with DESeq2. Genome Biol 2014;15:550.

30. Carlson CS, Emerson RO, Sherwood AM, et al. Using synthetic templates to design an unbiased multiplex PCR assay. Nat Commun 2013;4:2680.

31. Sánchez-Magraner L, Miles J, Baker CL, et al. High PD-1/PD-L1 Checkpoint Interaction Infers Tumor Selection and Therapeutic Sensitivity to Anti-PD-1/PD-L1 Treatment. Cancer Research 2020;80:4244.

32. Thorsson V, Gibbs DL, Brown SD, et al. The Immune Landscape of Cancer. Immunity 2018;48:812-830 e14.

33. Le DT, Durham JN, Smith KN, et al. Mismatch repair deficiency predicts response of solid tumors to PD-1 blockade. Science 2017;357:409-413.

34. Repana D, Nulsen J, Dressler L, et al. The Network of Cancer Genes (NCG): a comprehensive catalogue of known and candidate cancer genes from cancer sequencing screens. Genome Biol 2019;20:1.

35. Turajlic S, Litchfield K, Xu H, et al. Insertion-and-deletion-derived tumour-specific neoantigens and the immunogenic phenotype: a pan-cancer analysis. Lancet Oncol 2017;18:1009-1021.

36. Ballhausen A, Przybilla MJ, Jendrusch M, et al. The shared neoantigen landscape of MSI cancers reflects immunoediting during tumor evolution. Preprint at: https://www.biorxiv.org/content/10.1101/691469v1 2019.

37. Feng M, Jin JQ, Xia L, et al. Pharmacological inhibition of beta-catenin/BCL9 interaction overcomes resistance to immune checkpoint blockades by modulating Treg cells. Sci Adv 2019;5:eaau5240.

38. Coelho MA, de Carne Trecesson S, Rana S, et al. Oncogenic RAS Signaling Promotes Tumor Immunoresistance by Stabilizing PD-L1 mRNA. Immunity 2017;47:1083-1099 e6.

39. Charoentong P, Finotello F, Angelova M, et al. Pan-cancer Immunogenomic Analyses Reveal Genotype-Immunophenotype Relationships and Predictors of Response to Checkpoint Blockade. Cell Rep 2017;18:248-262.

40. Zhao J, Chen AX, Gartrell RD, et al. Immune and genomic correlates of response to anti-PD-1 immunotherapy in glioblastoma. Nat Med 2019;25:462-469.

41. Grasso CS, Giannakis M, Wells DK, et al. Genetic Mechanisms of Immune Evasion in Colorectal Cancer. Cancer Discov 2018;8:730-749.

42. Rizvi NA, Hellmann MD, Snyder A, et al. Cancer immunology. Mutational landscape determines sensitivity to PD-1 blockade in non-small cell lung cancer. Science 2015;348:124-8.

43. Sade-Feldman M, Jiao YJ, Chen JH, et al. Resistance to checkpoint blockade therapy through inactivation of antigen presentation. Nat Commun 2017;8:1136.

44. McGranahan N, Rosenthal R, Hiley CT, et al. Allele-Specific HLA Loss and Immune Escape in Lung Cancer Evolution. Cell 2017;171:1259-1271 e11.

45. Middha S, Yaeger R, Shia J, et al. Majority of B2M-Mutant and -Deficient Colorectal Carcinomas Achieve Clinical Benefit From Immune Checkpoint Inhibitor Therapy and Are Microsatellite Instability-High. JCO Precis Oncol 2019;3.

46. De Nicola F, Goeman F, Pallocca M, et al. Deep sequencing and pathway-focused analysis revealed multigene oncodriver signatures predicting survival outcomes in advanced colorectal cancer. Oncogenesis 2018;7.

47. Castro A, Ozturk K, Pyke RM, et al. Elevated neoantigen levels in tumors with somatic mutations in the HLA-A, HLA-B, HLA-C and B2M genes. BMC Med Genomics 2019;12:107.

48. Burr ML, Sparbier CE, Chan KL, et al. An Evolutionarily Conserved Function of Polycomb Silences the MHC Class I Antigen Presentation Pathway and Enables Immune Evasion in Cancer. Cancer Cell 2019;36:385-401 e8.

49. Demaria O, Cornen S, Daeron M, et al. Harnessing innate immunity in cancer therapy. Nature 2019;574:45-56.

50. Pardoll DM. The blockade of immune checkpoints in cancer immunotherapy. Nat Rev Cancer 2012;12:252-64.
